# Electron Ionization of Imidazole and Its Derivative 2-Nitroimidazole

**DOI:** 10.1007/s13361-019-02337-w

**Published:** 2019-10-30

**Authors:** Rebecca Meißner, Linda Feketeová, Anita Ribar, Katharina Fink, Paulo Limão-Vieira, Stephan Denifl

**Affiliations:** 1 Institut für Ionenphysik und Angewandte Physik and Center for Molecular Biosciences Innsbruck (CMBI), Universität Innsbruck, Technikerstraße 25, 6020 Innsbruck, Austria; 2 Atomic and Molecular Collisions Laboratory, CEFITEC, Department of Physics, Universidade NOVA de Lisboa, 2829-516 Caparica, Portugal; 3 Institut de Physique des 2 Infinis de Lyon; CNRS/IN2P3, UMR5822, Université de Lyon, Université Claude Bernard Lyon 1, 43 Bd du 11 novembre 1918, 69622 Villeurbanne, France

**Keywords:** Electron ionization, Imidazole, Nitroimidazole, Appearance energy, Gas phase, Transition state, Unimolecular dissociation

## Abstract

Imidazole (IMI) is a basic building block of many biologically important compounds. Thus, its electron ionization properties are of major interest and essential for the comparison with other molecular targets containing its elemental structure. 2-Nitroimidazole (2NI) contains the imidazole ring together with nitrogen dioxide bound to the C2 position, making it a radiosensitizing compound in hypoxic tumors. In the present study, we investigated electron ionization of IMI and 2NI and determined the mass spectra, the ionization energies, and appearance energies of the most abundant fragment cations. The experiments were complemented by quantum chemical calculations on the thermodynamic thresholds and potential energy surfaces, with particular attention to the calculated transition states for the most important dissociation reactions. In the case of IMI, substantially lower threshold values (up to ~ 1.5 eV) were obtained in the present work compared to the only available previous electron ionization study. Closer agreement was found with recent photon ionization values, albeit the general trend of slightly higher values for the case of electron ionization. The only exception for imidazole was found in the molecular cation at *m*/*z* 40 which is tentatively assigned to the quasi-linear HCCNH^+^/ HCNCH^+^. Electron ionization of 2NI leads to analogous fragment cations as in imidazole, yet different dissociation pathways must be operative due to the presence of the NO_2_ group. Regarding the potential radiosensitization properties of 2NI, electron ionization is characterized by dominant parent cation formation and release of the neutral NO radical.

**Electronic supplementary material:**

The online version of this article (10.1007/s13361-019-02337-w) contains supplementary material, which is available to authorized users.

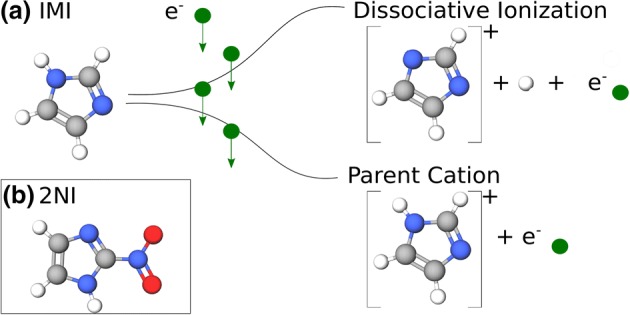

## Introduction

**Scheme 1:**
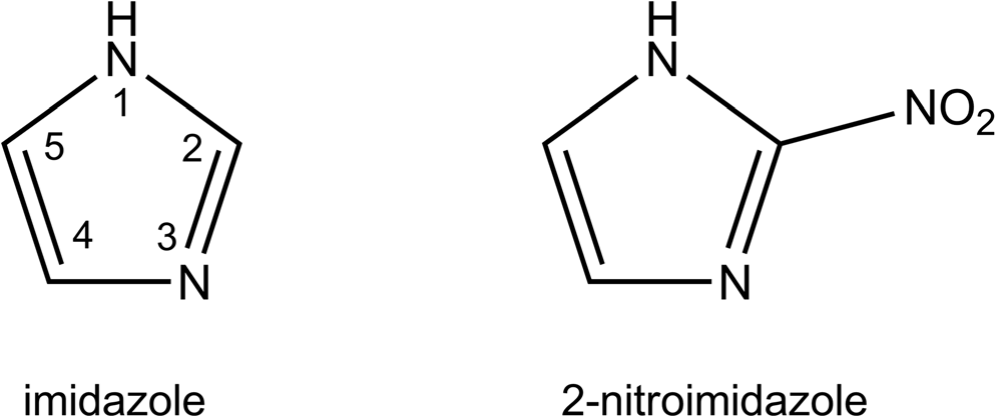
Structures of imidazole (left) and 2-nitroimidazole (right)

Radiation is a major source of damage for intact cells [1, 2]. It is widely used in radiotherapy to kill tumor cells. The drawback of the radiation is that also healthy tissue may be damaged. The lack of selectivity is addressed by radiosensitizers, increasing the ratio of damage of malignant to healthy cells [3, 4]. One problem is to overcome hypoxia, which is common in many cancerous tissues [5, 6]. The deprival of oxygen results in a change of metabolism and additionally, those cells show a higher resistance towards radiation [7]. Drugs substituting deprived oxygen in tumor cells are known as oxygen mimetic [5]. Nitric oxide was shown to act highly efficient, even surpassing the effects of oxygen itself [8–12]. 2-Nitroimidazole (2NI) (see Scheme 1) is based on imidazole (IMI). It belongs to the class of nitroimidazole compounds, which have been proven beneficial as oxygen mimetic radiosensitizer in clinical trials and are further used as antibacterial drugs and in antibiotics [11, 13–15]. Imidazole itself is a basic building block in biology, present in the amino acid histidine, the hormone histamine, and the nucleobase purine. It thus serves as model compound for more complex molecular systems present in different environments with particular attention to the biological mediums. It is well established that the physical and chemical properties of a molecule define its role in determining the local interaction with its surrounding molecules. Thus, a detailed knowledge of the chemical and physical properties of IMI and 2NI can help creating novel compounds with relevant impact for medical use and other scientific and even technological applications given their thermal stability, high heats of formation, and good detonation performance [16].

As imidazole is an omnipresent DNA/RNA building block, the ionization energy and fragmentation pathways have been investigated with different methods. Photoelectron spectroscopy (PES) studies provided information about the electronic structure and ionization energy of IMI [17–20] with values similar to the theoretical study of Cuong et al. [21]. Further spectroscopy investigations reported on the electron binding energy of the IMI anion [22], on the vertical electron attachment energy [23], and on the anionic deprotonation pathway [24]. The latter investigation was recently extended by a study of multiple hydrogen loss reactions in neutral IMI upon dissociative electron attachment [25]. Additionally, we note the study from Klebe et al., addressing the loss of the hydrogen atom and the hydrogen cyanide molecule upon electron ionization and reporting the appearance energies of cationic fragments [26]. Later, and apart from the ionization energy, Main-Bobo et al. extended the study by photoionization mass spectrometry and photoelectron photoion coincidence spectroscopy data to the appearance energies of some fragment cations [27]. Another mass-spectrometric photoionization study of Schwell et al. completed this data, proposing fragmentation pathways based on the experimental and calculated values by determining the ionization and appearance energies [28]. Nevertheless, the present study is crucial to examine Main-Bobo et al. findings since electron ionization and photoionization cause a striking different behavior in some fragmentation channels of IMI due to the possibility to excite states by electron impact which are not accessibly by photon excitation [27]. Here we present a complete study of the cationic fragments of IMI upon electron ionization and compare the appearance energies with Klebe et at. [26], improving significantly the precision of the appearance energies and extending the total number of observed fragment channels. Additionally, these results are compared with those obtained from photoionization studies [27, 28].

Similarly, nitroimidazoles have been studied by PES to determine their electronic structure [19, 29, 30]. Decomposition products upon photon excitation were reported in [31] with nitric oxide (NO) and nitrogen dioxide (NO_2_) as the main fragments. A recent photofragmentation study of doubly charged, core excited 4(5)-nitromindazole (4(5)-NI) showed that methylation at the N1 position (see Scheme 1) suppresses efficiently the NO and NO^+^ production [32]. The first mass spectra of electrosprayed nitroimidazolic compounds, like nimorazole, were recorded by Feketeová et al. [33, 34]. They also determined product ions of collision-induced and electron-induced dissociation. Additionally, the isomers 2-NI and 4(5)-NI were studied by dissociative electron attachment experiments; revealing isomer effects regarding the position of the NO_2_ group [35] and bond breaking selectivity was achieved upon methylation at the N1 position [36] as well as in electron transfer experiments [37]. Besides, valence ionization of nitroimidazoles was investigated by photoelectron–photoion coincidence (PEPICO) measurements [38]. Recently, two photofragmentation studies investigated the fundamental mechanisms of the bond-breaking, also reporting the ionization and appearance energies of the cationic parent and fragment products [39, 40]. These experimental results were supported by calculations of the fragmentation pathways [40]. In the present study, we also report fragmentation reactions of 2-NI upon electron ionization, derive for the first time ionization and appearance energies, and compare them with the available photoionization studies.

**Figure 1:**
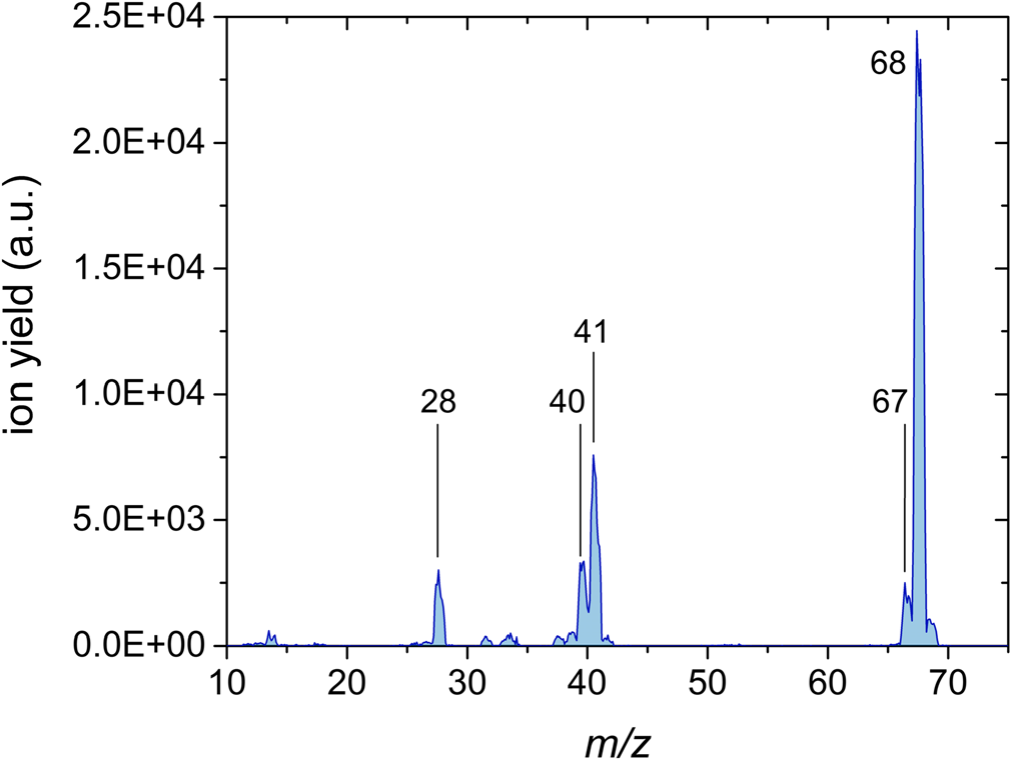
Mass spectrum of imidazole obtained by electron ionization at the electron energy of 70 eV

**Figure 2:**
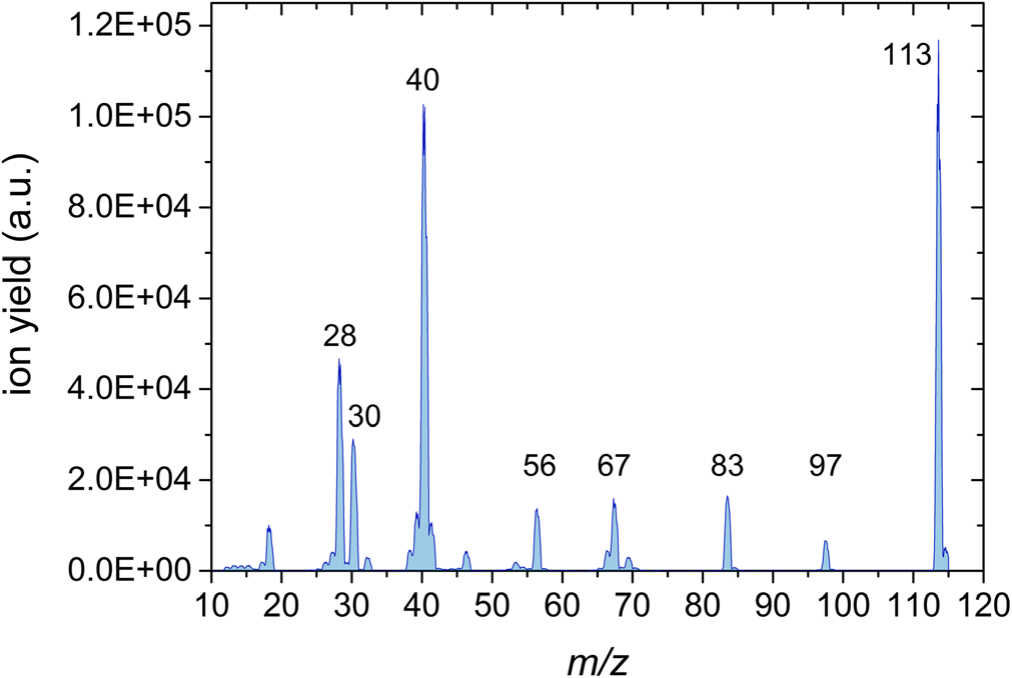
Mass spectrum of 2-nitroimidazole obtained by electron ionization at the electron energy of 70 eV

In addition to ionization and appearance energies of IMI and 2NI, mass spectra at the electron energy of 70 eV are presented (Figures 1 and 2) in order to elucidate the most important fragmentation pathways. The experimental results are supported by quantum chemical calculations determining the thermodynamic thresholds of dissociation reactions for both molecular compounds. Finally, for IMI, we also explored potential energy surface and calculated transition states for the most important dissociation reactions, and for 2NI, we propose pathways for the formation of the fragment ion due to the loss of the –NO_2_ group, which remained an opened question in the previous study using photoionization.

## Experimental Methods

The experimental setup utilized for the present studies consists of a quadrupole mass spectrometer coupled with a high resolution electron monochromator, described in detail elsewhere [41]. Both IMI and 2NI were purchased from Sigma-Aldrich with stated purities of 99.5% and 98%, respectively and were used as delivered.

The compounds under investigation were placed inside of an oven inside of the vacuum chamber. At the typical experimental base pressures of about 10^−9^–10^−8^ mbar, heating to about 100 °C was required for both compounds in order to achieve sufficient sublimation. An effusive beam of molecules was formed by a capillary with 1 mm diameter which was mounted onto the oven. A hemispherical electron monochromator (HEM) generated an energetically and spatially focused electron beam with a resolution of about 100 meV. The stability of the electron beam was constantly monitored by a pico-ampere meter connected with a Faraday cup, which was placed after the crossing zone with the neutral beam. In the interaction region, located within the HEM right before the Faraday cup, the IMI or 2NI was ionized. In order to analyze the ionic products, a weak electrostatic field extracted them into a quadrupole mass analyzer with nominal mass range of 2048 u. The mass selected ions were detected with a channel type secondary electron multiplier operated in pulse counting mode and processed by a combined preamplifier and detection unit.

All ionization efficiency curves were measured within an energy region of minimum ± 1.5 eV around the ionization/appearance energy. For calibration of the energy scale, the ionization efficiency curve of helium or neon was measured and compared to the well-known literature values of 24.587 eV and 21.565 eV, respectively [42].

The statistical uncertainty of the threshold values discussed in the following sections is composed of the shift of the AE value caused by the choice of the input parameter and the uncertainty of the fit. The systematic error is caused by the uncertainty on the neon or helium calibration and amounts to 0.01 eV.

## Computational Methods

### Determination of Ionization and Appearance Energies

The experimental ionization and appearance energies (IE and AE) were determined by fitting a potential function in a similar form as suggested from Wigner [43] and Wannier [44] for the theoretical ionization cross section close to threshold. Wigner described the cross section behavior at threshold by a simple power law, which depends on the number of outgoing electrons in the exit channel. Specifically for single electron ionization, a linear rise in the cross section was predicted for single ionization processes [43]. Wannier further specified the model to a final state including one ion and two electrons [44], resulting in the function

1 $$ f(E)=b+c\cdot {\left(E-\mathrm{AE}\right)}^n\cdot \theta \kern0.15em \left(E-\mathrm{AE}\right) $$

with *b* the background, *c* a scaling parameter, *E* the electron energy, AE the appearance or ionization energy, and *θ* the Heaviside function which is required to model the behavior below threshold. The exponent *n* was only calculated for hydrogen so far and is in all other cases issue to experimental determination.

Since the experimental setup is subject to a finite energy resolution, we convoluted function *f*(*E*) with a Gaussian function to account for the experimental conditions. Thus, the function fitted to the data was

2 $$ \sigma (E)=b+\frac{c}{\sqrt{2\pi }}{\rho}^n\cdot \Gamma \kern0.15em \left(n+1\right)\cdot \exp \kern0.15em \left(-\frac{1}{4{\rho}^2}{\left(\mathrm{AE}-E\right)}^2\right)\cdot {D}_{n+1}\left(\frac{1}{\rho}\left(\mathrm{AE}-E\right)\right) $$

with Γ the gamma function, *D*_*n* + 1_ a parabolic cylinder function, and *ρ* the standard deviation of the Gaussian and energy resolution of the HEM.

Some ionization efficiency curves featured two onsets. In this case, the fitting function was extended by adding a second power law including a Heaviside function to Eq. (1) and convoluting this formula as for single onsets.

The analysis software, based on a previous version [45], was written in Python using the SciPy, NumPy, and Matplotlib libraries. The required input parameters included the starting values for the free parameters and the fitting range. Then, the ionization efficiency curves were fitted by means of the Chi-square method and as most important output parameter, the appearance and ionization energies were derived.

### Quantum Chemical Calculations

Quantum chemical calculations employing M062x/aug-cc-pVTZ level of theory [46, 47] and basis set [48] were carried out with the Gaussian-09D01 program package [49] to calculate vertical ionization energies (VIE), adiabatic ionization energies (AIE), and the free energy of reactions, ∆*G.* Such energy is calculated for each fragmentation pathway as Δ*G* = Σ*G*(products) − Σ*G*(reactants), where in the currently studied electron ionization processes, the reactant refers to neutral IMI or 2NI.

For all structures, the frequencies were calculated to confirm that those are local minima on the potential energy surface. All energies were corrected for zero-point energies. Transition states (TS) for fragmentation pathways were optimized at the same level of theory and basis set. The frequencies of TS were calculated to confirm that the structures are local maxima on the potential energy surface. Calculations of the intrinsic reaction coordinates (IRC) connected the TS to reactants and products. We estimate an error of less than 2 kcal mol^−1^ (0.09 eV) for the free energies of the reactions from the reported mean unassigned error for M062x thermochemistry [46]. All the considered structures, dissociation pathways, and an example of IRC calculation are summarized in the Electronic Supplementary Material (ESM).

## Results and Discussion

### Mass Spectra

Electron ionization (EI) is rather a hard ionization method in which an energetic electron interacts with a molecule removing an electron from the neutral compound. Depending on the excess energy deposited, either the parent cation stabilizes or it dissociates into one positively charged fragment and one or more neutral fragments. The ionization method may influence the observed mass spectrum. The possibility of different fragment ions’ formation was mentioned by Main-Bobo et al. [27]. The transmission of the mass analyzer and ion collection efficiency can further cause deviations in relative peak intensities, but the number of observed fragments should not be altered. Hence, recording the ionization mass spectra of IMI and 2NI allows the assignment of the cationic fragments and a comparison with photoionization studies [27, 28, 39, 40], together with EI spectra from the NIST database [50] and the Spectral Database for Organic Compounds (SDBS) [51].

#### Imidazole

In the case of the ionization mass spectrum of imidazole (see Figure 1), the most intense ion is assigned to the parent cation at *m*/*z* 68. This agrees well with the NIST database [50] but represents the major difference to the photoionization mass spectrum recorded at 21 eV by Schwell et al. [28], where the parent cation is very weakly abundant. In contrast, the two most intense ions were reported at *m*/*z* 41 and *m*/*z* 40, assigned to C_2_H_3_N^+^ and C_2_H_2_N^+^, respectively. In the present mass spectrum, these ions form the most dominant fragments, where C_2_H_3_N^+^ yields one third the intensity of the parent cation. This cation is formed by HCN loss which was also reported by Klebe at al. [26]. Additionally, two other cationic fragments are observed with considerable yields, *m*/*z* 28 assigned to HCNH^+^ and at *m*/*z* 67 assigned to [IMI − H]^+^, which corresponds to the dehydrogenated parent cation. Some further weak fragment ions as well as isotopic contributions of abundant fragment ions are also visible in the spectrum. They are of small intensities, and therefore, no appearance energies were determined. From the general point of view, the ionization mass spectrum agrees well with the EI spectrum from NIST [50], Klebe et al. [26], and Schwell et al. [28]. Only as far as the parent cation is concerned, the present yield differs from [28]. Notwithstanding, these authors noted that the low intensity of the parent cation resulted from mass discrimination properties of the quadrupole mass analyzer used in their experiments.

#### 2-Nitroimidazole

The ionization mass spectrum of 2NI (see Figure 2) reveals, as expected, some similarities in the product ions when compared to imidazole. Again, the parent cation at *m*/*z* 113 possesses the strongest yield. In contrast to IMI, no dehydrogenated parent cation is observed. With comparably high intensity, the fragment at *m*/*z* 40 is assigned to HCCNH^+^, where losses of NO_2_ and HCN from the parent cation are operative. The third most intense cation at *m*/*z* 28 is assigned to HCNH^+^. While for the IMI the fragment ion at *m*/*z* 40 is half of the feature at *m*/*z* 41, in the case of 2NI the latter is strongly suppressed. At *m*/*z* 67, we report C_3_H_3_N_2_^+^, which is formed by the loss of the NO_2_ group and is assigned to [IMI – H]^+^. The peaks at *m*/*z* 30, 56, 83, and 97 all contain contributions from the nitrogen dioxide group. Here, *m*/*z* 30 (NO^+^) and *m*/*z* 83 (C_3_H_3_N_2_O^+^) represent counterparts and stem from the same bond breaking. For *m*/*z* 56, additionally to the cleavage of the nitro group, HCN loss from the imidazole ring occurs, a process similar to the formation of the fragment cation at *m*/*z* 40. At *m*/*z* 97, we report C_3_H_3_N_3_O^+^ that corresponds to the release of a neutral oxygen atom from the parent cation. Comparing the present mass spectrum to [51], the photoionization data of Bolognesi et al. [39] and Cartoni et al. [40], the detected fragments are identical albeit with different relative peak intensities (which may also be related to different experimental conditions). For example, in the SDBS spectrum [51], the prominent *m*/*z* 40 peak is a factor of ~1.7 higher than the parent cation, while here and in the work of Cartoni et al. [40], the intensity of both ions is similar. Bolognesi et al. [39] report a strongly suppressed parent cation and a higher number and abundance of fragment cations. A recent PEPICO study of 2NI by Itälä et al. [38] also reported the mass spectrum at the photon energy of 16 eV. The spectrum featured the parent cations, NO^+^ and [2NI − NO]^+^, at *m*/*z* 83 as the strongest channels [38].

### Ionization and Appearance Energies

The ionization efficiency curves near threshold were measured for the most abundant cations in the mass spectra. The experimental data were fitted by the method described above to determine the experimental ionization and appearance energies. Subsequently, the results are presented, discussed, and compared with both calculations and earlier studies.

#### Imidazole

**Figure 3:**
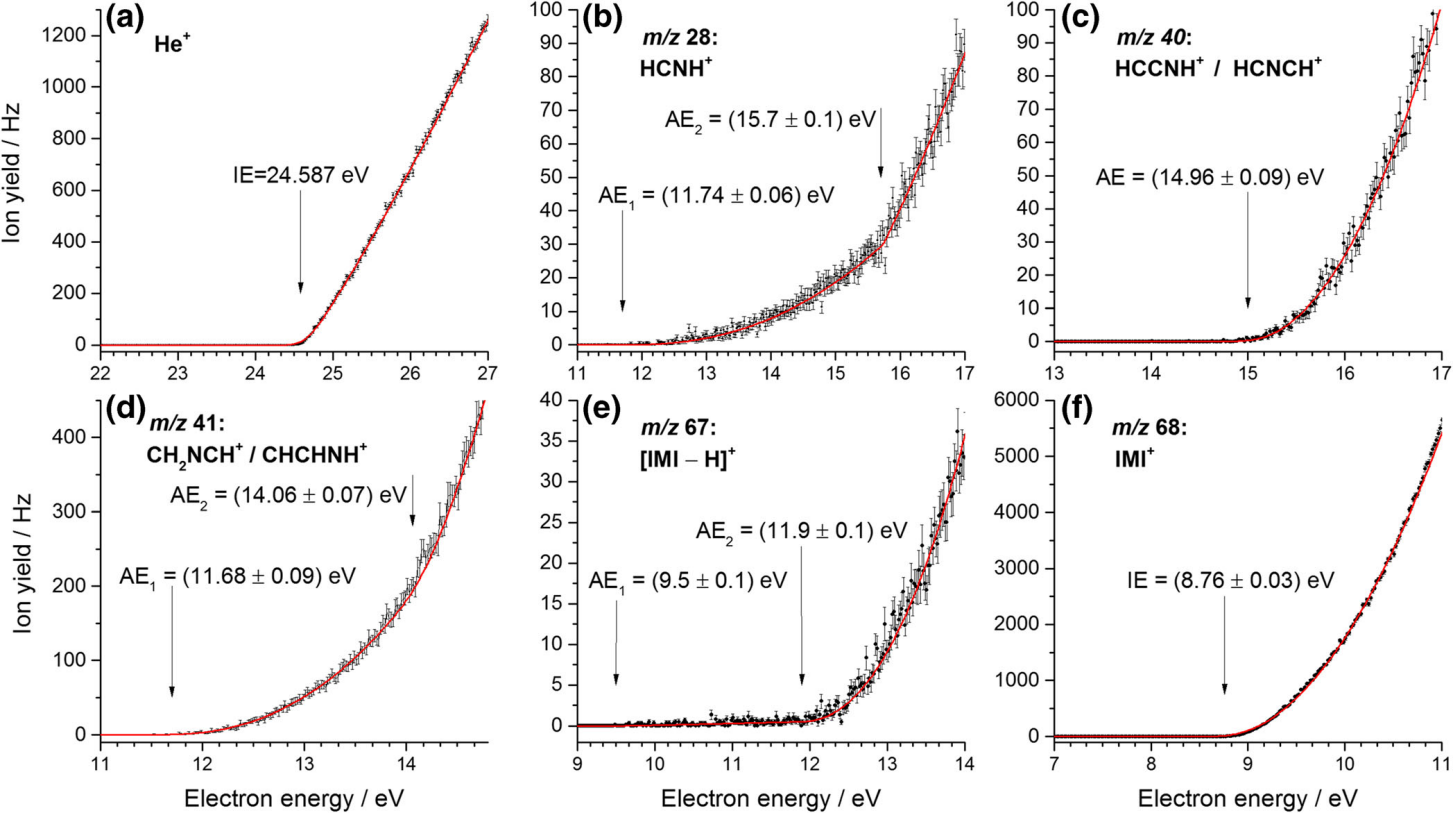
Threshold ionization efficiency curves of imidazole (**b**–**f**). The data is shown as black dots, including the statistical uncertainties as error bars. The red solid lines represent the fitted functions. For each cation, the determined AE is indicated by a black arrow. (**a**) The threshold ionization efficiency of helium which was used for calibration

For IMI, the experimental data and fits are shown in Figure 3 and summarized in Table 1. The energy scale was calibrated by measuring the ion yield curve of helium and determining its onset of formation (see Figure 3a). The mass spectrum indicates that the parent cation forms the most intense channel upon EI of imidazole. We report an experimental ionization energy of 8.76 ± 0.03 eV (see Figure 3f) which is slightly lower than the calculated adiabatic ionization energy (AIE) of 8.83 eV. The vertical ionization energy is also calculated and amounts to 9.08 eV. Calculations on the ring opening (Figure ESM_1 in ESM) and hydrogen transfer (Figure ESM_2) in the ionized IMI^+^ cation suggest barriers > 1.69 eV and > 2.39 eV, respectively. Thus, these processes are not possible in the parent cation formed close to threshold. The earlier reported values of the ionization energy of IMI cover a range from 8.66 eV (VUV study of Schwell et al. [28]) up to 9.12 eV (EI study of Klebe et al. [26], who utilized a double-focusing mass spectrometer with a standard ion source, i.e., no monochromatizing element for the electron beam, in contrast to the present study) [17–21, 27].

**Table 1:** Summary of Observed Cations Upon Electron Ionization of Imidazole, Including *m*/*z* Value, Assigned Cation and Experimental and Calculated Ionization and Appearance Energy Values, Together with Available Literature Values. All the Calculated Values Refer to the Singlet States of the Respective Cations Unless Marked

*m*/*z*	Assignment		IE and AE values (eV)		Previous EI exp.	Previous PI exp.
	Cation	Neutral	Present exp.	Present calc.		
28	HCNH^+^	CH_2_CN CH_2_NC	11.74 ± 0.06 11.74 ± 0.06	11.65 11.74		11.67 ± 0.05^a^, 11.34 ± 0.05^b^
40	HCCNH^+^ HCNCH^+^	HCNH HCN + H	14.96 ± 0.09 14.96 ± 0.09	15.09 15.07*		13.83 ± 0.05^b^
41	CH_2_NCH^+^ CH_2_CNH^+^ CHCHNH^+^	HCN HCN HCN	11.68 ± 0.09 11.68 ± 0.09 14.06 ± 0.07	11.80 11.38 14.02	13.2^c,d^	11.48 ± 0.02^a^, 11.41 ± 0.05^b^
67	[IMI − H]^+^	H	11.9 ± 0.1	11.98, 12.03, 12.04	12.8^c,d^	11.38 ± 0.05^b^, 12.05 ± 0.03^b^
68	IMI^+^		8.76 ± 0.03	8.83 (AIE)	9.12^c^	8.66–8.96^e^

*Calculated value refers to the triplet state of respective cation

^a^Refer to reference [27]. Their assignment to a specific cationic and neutral structure can be found in the same line, see columns cation and neutral

^b^Refer to reference [28]. Their assignment to a specific cationic and neutral structure can be found in the same line, see columns cation and neutral

^c^Refer to reference [26]

^d^No assignment of the AE to a specific cationic and neutral structure was reported

^e^The range refers to values reported in the references [17–20, 27, 28]

**Figure 4:**
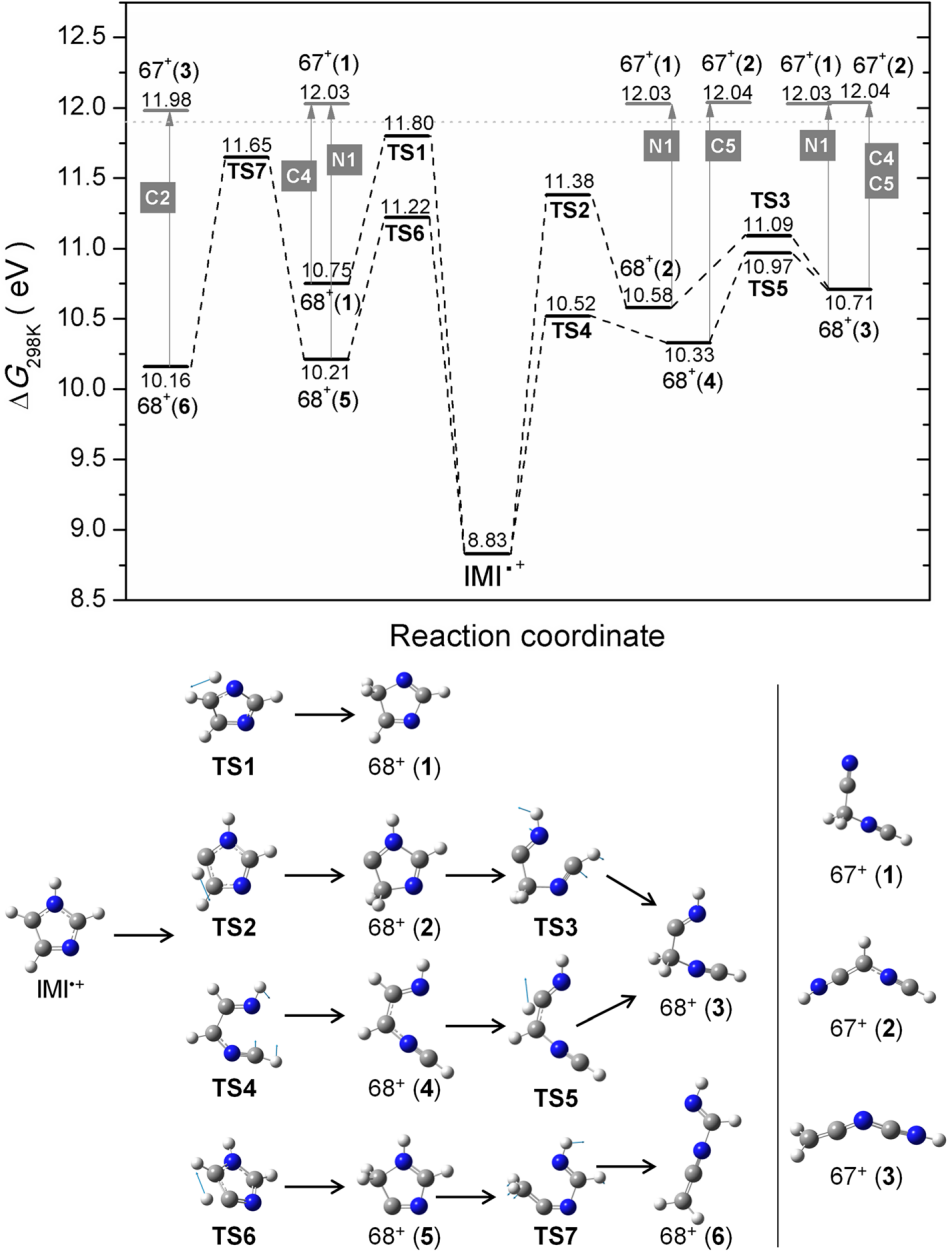
M06-2x/aug-cc-PVTZ calculated potential energy diagram for the decomposition of the IMI^+^ leading to the formation of [IMI − H]^+^ fragment at *m*/*z* 67 including associated structures shown below. The blue arrows in the respective TS show the displacement vectors. The labels in a gray square refer to the position of the imidazole ring from which the H has been lost. The dotted line corresponds to the experimental value

The combination of the parent cation exhibiting a very high intensity compared to the [IMI − H]^+^ fragmentation channel and the use of a quadrupole mass spectrometer with limited mass resolution leads to a weak contamination of the ion efficiency curve of [IMI − H]^+^ which shows two onsets (see Figure 3e). The first onset at 9.5 ± 0.1 eV can be assigned to a background contribution from the IMI^+^ ion yield. The disagreement with the IE mentioned above can be explained by the very low ion yield below the second onset. In contrast, the second observed onset is assigned to the appearance energy of [IMI – H]^+^ at 11.9 ± 0.1 eV. Earlier VUV and EI studies reported values of 11.38 and 12.05 eV and 12.8 eV, respectively [26, 28]. All the calculated [IMI − H]^+^ ions including triplet states are shown in Figure ESM_3. Based on the thermodynamic threshold, only several ring opened structures could be considered with the observed experimental onset of 11.9 ± 0.1 eV, i.e., no direct hydrogen loss seems to be operative. The H loss with a reverse barrier was already suggested by Main-Bobo et al. [27]. All calculated stable structures with intact ring are energetically not accessible (see Figure ESM_3). This contrasts the suggested cyclic [IMI − H]^+^ structures by Schwell et al. [28]. Experiments with deuterated IMI by Klebe et al. showed preferential (but not exclusive) hydrogen loss from the C4 and C5 positions [26]. Our calculations on H transfer in the ionized IMI (Figure ESM_2) show that H transfer mechanism is possible with ∆*G*(barrier) values in the range between 11.22 and 12.18 eV and thus are likely to play a role in the dissociation process. The calculated potential energy surface for H loss and the formation of linear [IMI − H]^+^ ions that are relevant to the observed experimental threshold are shown in Figure 4. Four pathways are shown. The H transfer from N1 position to C5 through TS1 of 11.80 eV results in cyclic ion 68^+^(**1**), where removal of H from C4 results in opening of the ring in the C2–N3 bond resulting in linear 67^+^(**1**) with ∆*G* of 12.03 eV. On the other hand, the H transfer from C5 to C4 through TS2 (11.38 eV) results in cyclic ion 68^+^(**2**), where removal of H from the N1 position leads to the opening of the ring and the formation of the same ion 67^+^(**1**). However, if the ion 68^+^(**2**) opens up the ring through TS3 (11.09 eV), it will lead to linear 68^+^(**3**), where the loss of H from the N1 position results in 67^+^(**1**), and the loss of H from the C4 results in 67^+^(**2**) with ∆*G* of 12.04 eV. In the latter, this would refer to the loss of H from the C4 or C5, which is the preferred loss observed by Klebe et al. [26]. Considering the dissociation of IMI^+^ starting with a ring opening reaction summarized in Figure ESM_1, only one such reaction showed to play a role below 12 eV. The ring opening between N1 and C2 through the TS4 (10.52 eV) leads to 68^+^(**4**), where a removal of H from the C5 results in 67^+^(**2**). For the ion 68^+^(**4**), further H transfer is possible from C5 to C4 through TS5 (10.97 eV) resulting in 68^+^(**3**) that, as mentioned above, through H loss can result in 67^+^(**1**) or 67^+^(**2**). Finally, H transfer from C4 to C5 through TS6 (11.22 eV) forms 68^+^(**5**), where removal of an H from N1 leads to the ring opening between C2 and N3 and the formation of 67^+^(**1**). If instead the ion 68^+^(**5**) opens up the ring between N1 and C5 through TS7 (11.65 eV), the linear ion 68^+^(**6**) is formed and H loss from the C2 position leads to the ion 67^+^(**3**) with ∆*G* of 11.98 eV. We note the observation of two thresholds by Schwell et al. [28] at 11.38 eV and a stronger at 12.05 eV, which can be related to two different TS involved in the dissociation rather than a loss of H from different positions.

**Figure 5:**
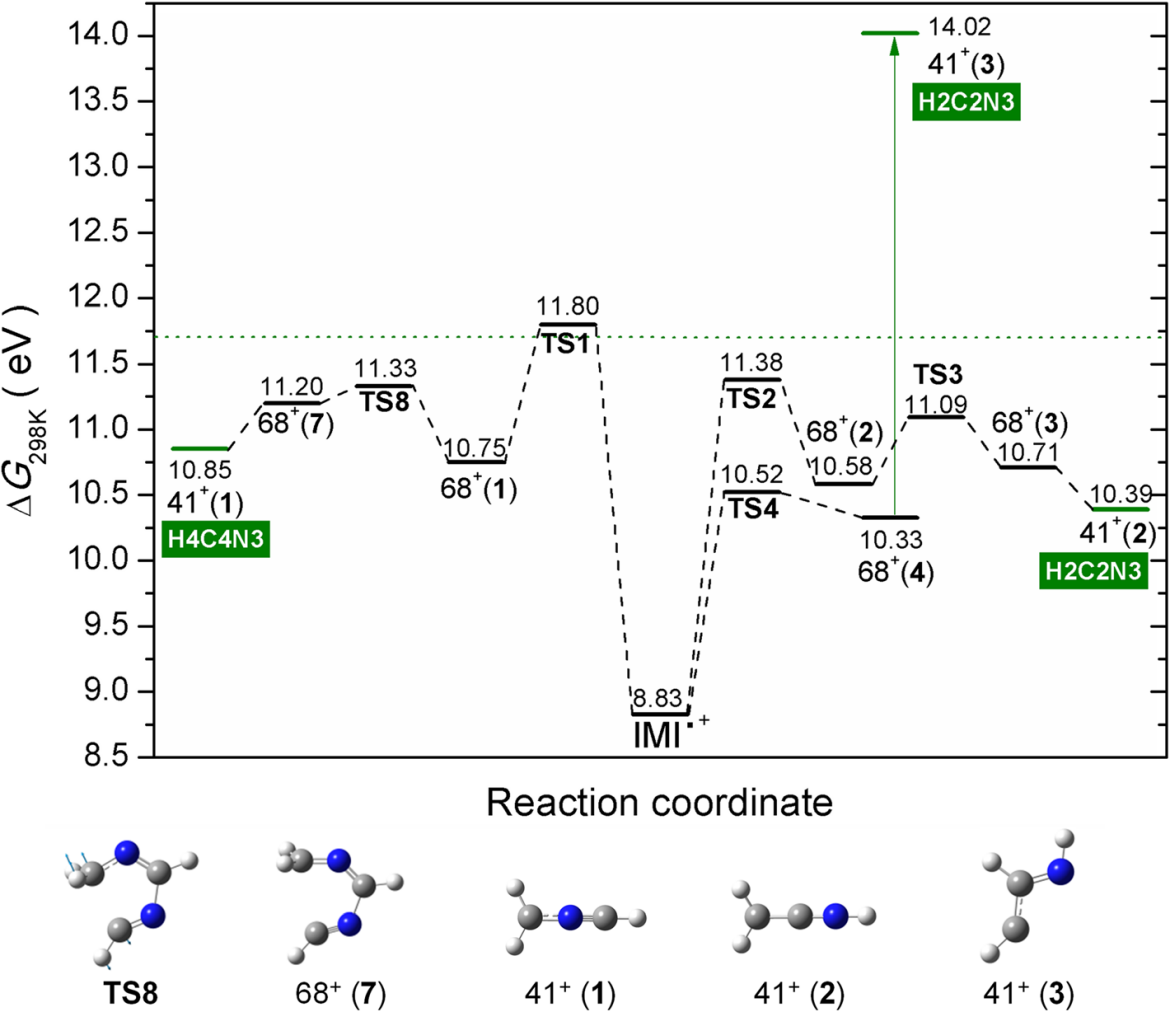
M06-2x/aug-cc-PVTZ calculated potential energy diagram for the decomposition of the IMI^+^ leading to the formation of fragment ion at *m*/*z* 41 including associated structures shown below. The blue arrows in the respective TS show the displacement vectors. The labels in a green rectangle refer to the position of the imidazole ring from which the HCN has been lost. The dotted line corresponds to the experimental value

The fragment ion C_2_H_3_N^+^ at *m*/*z* 41 can arise via multiple fragmentation pathways, leading to different assignments. Two experimental onsets are observed at 11.68 ± 0.09 eV and at 14.06 ± 0.07 eV (see Figure 3d). In the EI study by Klebe et al., an onset value of 13.2 eV is reported, while for the PI study by Main-Bobo et al. of 11.48 eV and for the VUV study by Schwell et al. of 11.41 eV [26–28]. Klebe et al. performed EI measurements with deuterated imidazoles and suggested that neutral HCN loss is preferred over HNC with preference of involving the C2 position and minor contributions of C4 and C5 [26]. Similarly, Main-Bobo et al. reported that HCN loss is the most likely, but with the addition that two fragmentation pathways are to be expected as they observed a bimodal kinetic energy release distribution in the metastable decay of the IMI parent cation on the microsecond time scale [27]. All the calculated structures considered in this study for the *m*/*z* 41 fragment ion are shown in Figure ESM_4. The potential energy surface for the formation of this ion is shown in Figure 5. It involves the same TS as mentioned above in the formation of the fragment ion at *m*/*z* 67. It seems reasonable considering the experimental thresholds being close in energy for the formation of these two ions. In Figure 5, the H transfer from N1 to C5 through TS1 (11.80 eV) results in cyclic 68^+^(**1**). The following ring opening between C5 and C4 through TS8 (11.33 eV) leads to 68^+^(**7**), where further breakage of the C2–N3 bond results in ion 41^+^(**1**) with ∆*G* of 10.85 eV. On the other hand, H transfer from C5 to C4 through TS2 (11.38 eV) followed by ring opening between N1 and C2 through TS3 (11.09 eV) leads to 68^+^(**3**), where further excision of the C4–N3 bond results in ion 41^+^(**2**) with ∆*G* of 10.39 eV. This is one of the pathways suggested by Main-Bobo et al. [27]. The ion 41^+^(**1**) is assigned to CH_2_NCH^+^ and a neutral HCN involves the C4 position, while 41^+^(**2**) is assigned to CH_2_CNH^+^ and a neutral HCN involves the C2 position. These fragmentation pathways are governed by the kinetics and thus in good agreement with the first observed threshold. Finally, the ring opening between N1 and C2 through TS4 (10.52 eV) leads to ion 68^+^(**4**), followed by a bond breaking between C4 and N3 that results in 41^+^(**3**) with ∆*G* of 14.02 eV. This value is in very good agreement with the second experimental threshold measured here. Our calculations show that the second pathway involves no hydrogen transfer. Again, the neutral fragment is HCN involving the C2 position. We just note that we also considered the pathways suggested by Schwell et al. [28], who reported the AE of 11.41 ± 0.05 eV for the formation of the ion at *m*/*z* 41. However, the suggestion on the initial rupture of the N3–C4 bond can be excluded due to the TS of 12.96 eV (see Figure ESM_1) that is additionally associated with H transfer from C5 to C4, where further bond breaking between N1 and C2 could also result in the formation of 41^+^(**2**), yet, with an apparent AE of 12.96 eV. The second pathway suggested by Schwell et al. [28] starts with the breakage of N1–C2 (related to our TS4; see Figure 4), followed by H transfer C5–C4 (related to TS5) and final breaking of the C4–N3 would lead to an apparent AE of 10.97 eV (TS5), which is too low. Our calculation shows that the H transfer must take place first and is followed by the ring opening as described above and as shown in Figure 5, where the TS2 (11.38 eV) would be in excellent agreement with the AE of 11.41 eV reported by Schwell et al. [28].

The fragment at *m*/*z* 40 has so far only been reported by Schwell et al. in a VUV study [28]. They reported an appearance energy of 13.83 ± 0.05 eV which is significantly lower than the experimentally determined onset in this study of 14.96 ± 0.09 eV (see Figure 3c). Thus, different dissociation pathways and products are possible. Schwell et al. discussed three possible fragmentation pathways for which we calculated all final states for both singlet and triplet states (see Figures ESM_5–ESM_7). The formation of the ion NCN^+^ can be excluded based on the thermodynamic threshold (see Figure ESM_5). The possible potential energy surfaces for the considered processes are shown in Figures ESM_8–ESM_10. Formation of the same fragment *m*/*z* 40, as a singlet in the VUV study and as a triplet in the EI study, can be excluded, as none of the possible structures shows the 1.13 eV difference expected for such two spin configurations (Figures ESM_5–ESM_7). Thus, it is more likely that a different structure of *m*/*z* 40 is formed by electron ionization compared to photon ionization. The first proposed pathway describes the loss of HCNH and/or CH_2_N from the parent cation IMI^+^. The structures are shown in Figure ESM_6, and the potential energy surface considered is shown in Figure ESM_8. However, it does not include formation of all structures present in Figure ESM_6. We considered the simplest path, i.e., breaking of two N–C bonds, followed by ring opening structures that can lead to loss of HCNH. However, these can all be excluded considering the study of Hodges et al. [52] on 1-methylimidazole with a deuterated methyl group showing that CD_3_ at the N1 position is not involved in the neutral loss. Thus, other pathways that we have not explored may be possible, like those involving H transfer reactions first before the N–C bond breaking. Based solely on the thermodynamic threshold (Figure ESM_6), the formation of quasi-linear HCCNH^+^ and HCNH gives a ∆*G* value of 15.09 eV that is in close agreement with our experiment.

The second suggested channel is the loss of HCN from [IMI − H]^+^. The structures with thermodynamic thresholds are summarized in Figure ESM_7, and the potential energy surface considered is shown in Figure ESM_9. Here, our calculations show that several fragmentation pathways are accessible and close to the experimentally observed threshold value. Loss of HCN from the 67^+^(**1**) yielding CH_2_CN^+^ is associated with ∆*G* of 14.77 eV, the loss of HCN from the 67^+^(**2**) results in HCCNH^+^ in a triplet state with a ∆*G* value of 14.68 eV, and finally the HNC loss from the 67^+^(**3**) giving CH_2_CN^+^ with ∆*G* of 15.23 eV. These pathways would be all in agreement with the studies on deuterated 1-methylimidazole by Hodges et al. [52]. However, the deviation from the present AE value of 14.96 ± 0.09 eV is too large.

The third pathway suggested by Schwell et al. is the loss of hydrogen from the *m*/*z* 41 cation. The structures with thermodynamic thresholds are summarized in Figure ESM_7, and the potential energy surface considered is depicted in Figure ESM_10. Depending on the position from which the hydrogen is stripped off, the 41^+^(**1**) could lead to the formation of CH_2_NC^+^ with ∆*G* of 14.73 eV, or formation of quasi-linear HCNCH^+^ in a triplet state with ∆*G* of 15.07 eV that is closest to our experimental value, while the 41^+^(**2**) can lead to the formation of the triplet HCCNH^+^ and the singlet CH_2_CN^+^ with calculated thresholds of 14.68 eV and 14.77 eV, respectively. However, the loss of H from the N position of 41^+^(**2**) can be excluded on the base of the 1-methylimidazole study by Hodges et al. [52]. The loss of H from C positions of 41^+^(**2**) and 41^+^(**3**) can form the same *m*/*z* 40 HCCNH^+^ in the triplet state with ∆*G* of 14.68 eV.

Thus, based on our calculations and the thermodynamic thresholds, we suggest that the fragment ion at *m*/*z* 40 is formed in electron collisions via loss of HCNH from IMI^+^ resulting in a quasi-linear ion HCCNH^+^ in the singlet state with ∆*G* of 15.09 eV and/or via the loss of HCN from 67^+^ and/or H loss from 41^+^ leading to a quasi-linear ion HCNCH^+^ in a triplet state with ∆*G* of 15.07 eV. Only one complete pathway has been identified here, namely the loss of H from *m*/*z* 41. Thus, we cannot exclude pathways that would be governed by the kinetics and not the thermodynamics, i.e., including TSs that we have not explored.

**Figure 6:**
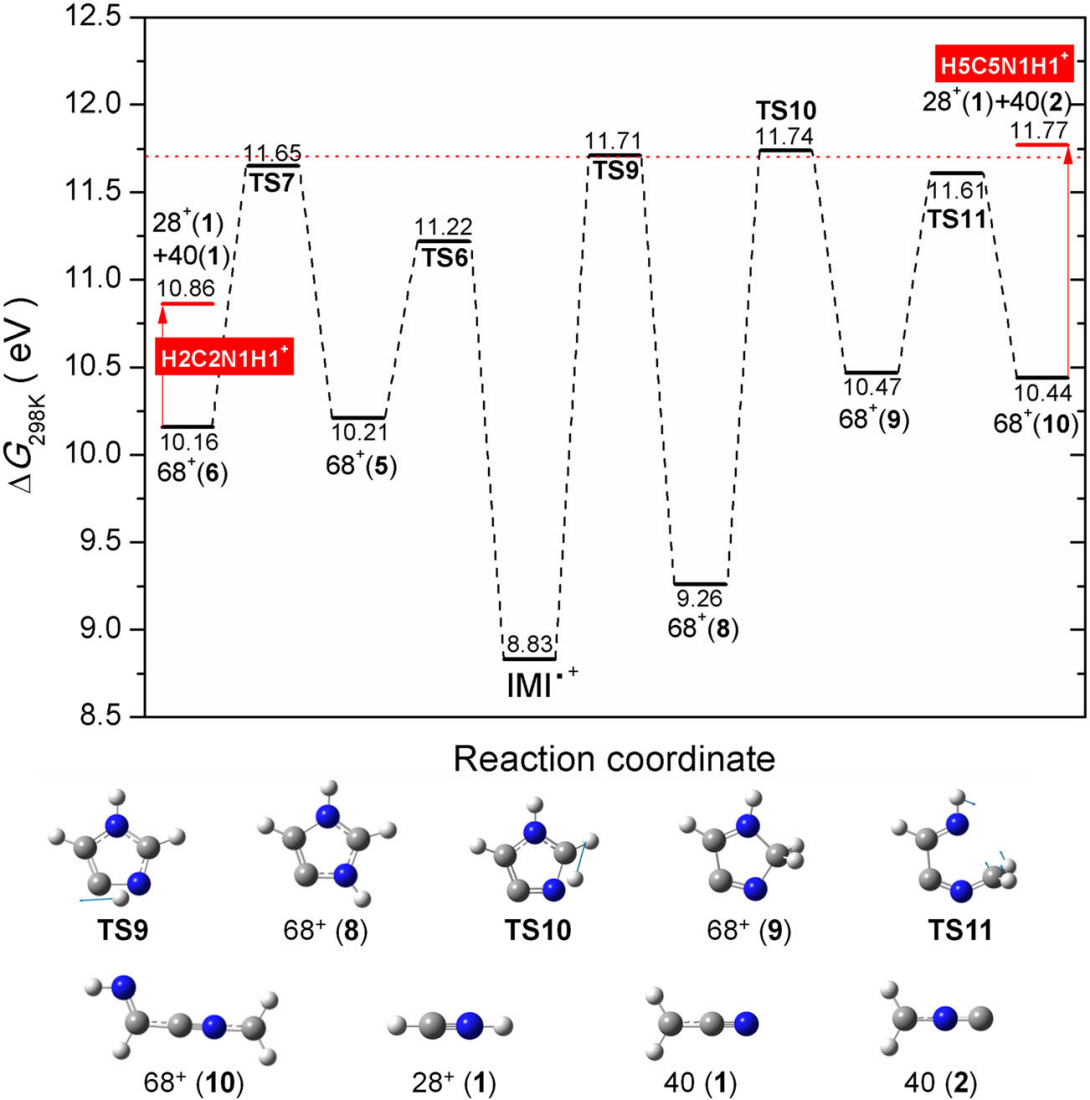
M06-2x/aug-cc-PVTZ calculated potential energy diagram for the decomposition of the IMI^+^ leading to the formation of fragment ion HCNH^+^ at *m*/*z* 28 including associated structures shown below. The blue arrows in the respective TS show the displacement vectors. The labels in a red rectangle refer to the position of the imidazole ring from which the ion *m*/*z* 28 has been formed. The dotted line corresponds to the experimental value

The measured ionization efficiency curve of *m*/*z* 28 reveals two onsets. In this context, it is worth noting that the N_2_^+^ cation cannot be separated from CH_2_N^+^ by its *m*/*z* due to the limited mass resolution of the quadrupole mass spectrometer. N_2_ exhibits an ionization energy of 15.6–15.7 eV as measured by earlier EI experiments [50] which matches our experimentally determined onset for the second threshold of 15.7 ± 0.1 eV (see Figure 3b). Thus, only the first onset is of interest for the fragmentation of imidazole. We derived a value of 11.74 ± 0.06 eV that is in closer agreement with 11.67 ± 0.05 eV reported by Main-Bobo et al. [27] than with the value of 11.34 ± 0.05 eV reported by Schwell et al. [28]. Based on thermodynamic reasons, Schwell et al. suggested that the neutral is lost as a single fragment [28]. We have considered different structures of the neutral (including formation of two neutrals) that are shown in Figure ESM_11. Figure 6 shows two fragmentation pathways, both possible from an energetic point of view. Although Main-Bobo et al. [27] suggested only one pathway based on the single component observed in the kinetic energy release distribution, the experiments with deuterated imidazole by Klebe et al. confirmed that both pathways suggested here are possible [26]. In the first fragmentation channel, which was also suggested by Main-Bobo et al. [27], a hydrogen is transferred from the C4 to the C5 position (TS6 11.22 eV). Subsequently, the N1–C5 bond breaks (TS7 11.65 eV) followed by the C2–N3 bond (10.86 eV). Finally, this results in the HCNH^+^ 28^+^(**1**) cation and a neutral 40(**1**) CH_2_CN fragment. In the second pathway, the hydrogen from the C4 position is first transferred to the N3 position (TS9 11.71 eV) forming 68^+^(**8**) and then further to the C2 position (TS10 11.74 eV) resulting in 68^+^(**9**). Afterwards, the N1–C5 bond breaks (TS11 11.61 eV), the ring opens yielding 68^+^(**10**), and the rupture of C4–C5 bond results in the HCNH^+^ 28^+^(**1**) cation and the neutral 40(**2**) CH_2_NC fragment. In both pathways, the resulting ion HCNH^+^ involves the N1 position, whereas the carbon atom involves the C2 in the first pathway and the C5 in the second pathway. Our experimental threshold is in excellent agreement with the calculated transition states involved in the dissociation process.

#### 2-Nitroimidazole

**Figure 7:**
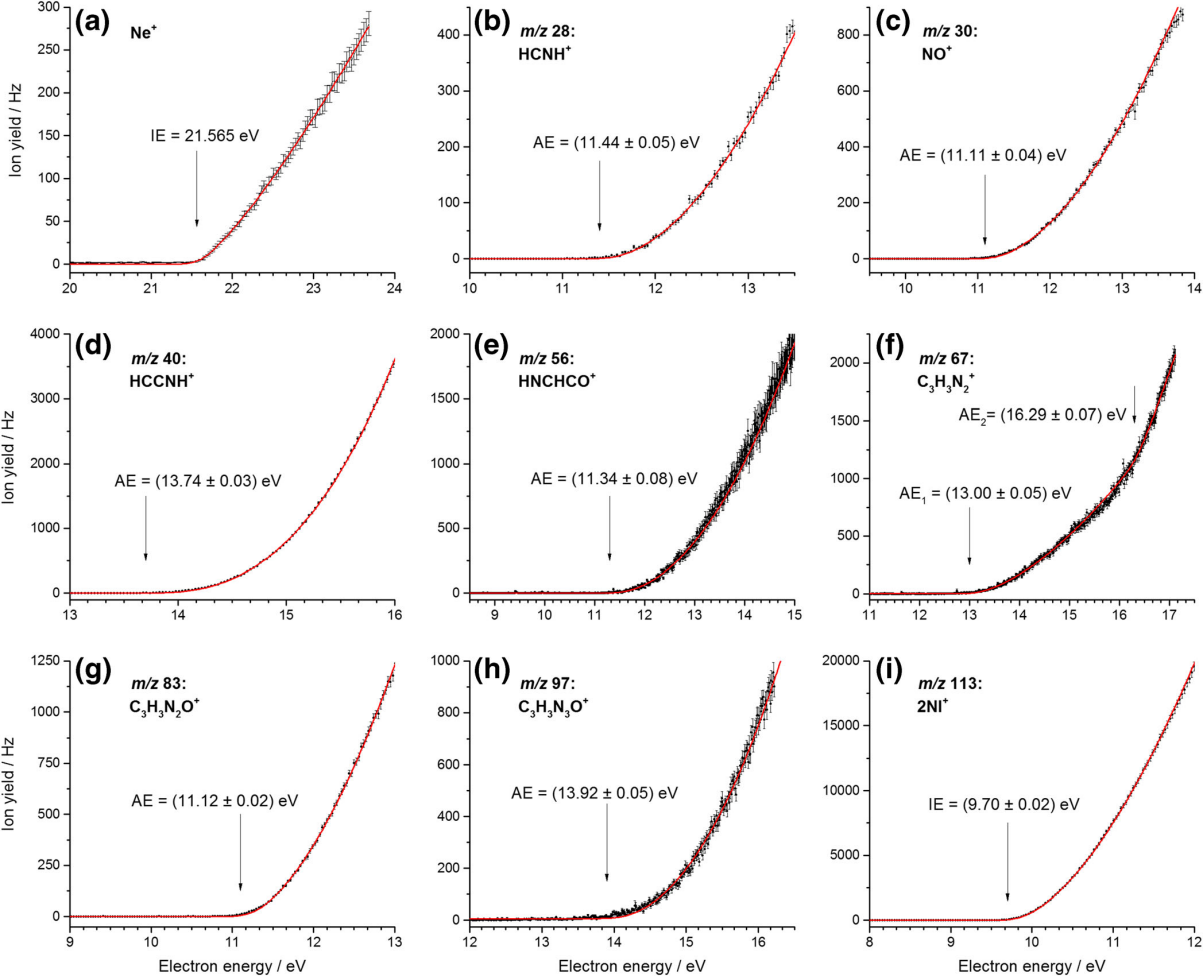
Threshold ionization efficiency curves of 2-nitroimidazole (**b**–**i**). The data is shown as black dots, including the statistical uncertainties as error bars. The red solid lines represent the fitted functions. For each cation, the determined AE is indicated by a black arrow. (**a**) The threshold ionization efficiency of neon which was used for calibration

For 2-nitroimidazole, the experimental threshold ionization efficiency data and fits are shown in Figure 7 and summarized in Table 2. Here, we used the well-known ionization energy of neon of 21.565 eV to calibrate the electron energy scale (see Figure 7a). Since Cartoni et al. already reported extensive calculations about the reaction mechanisms yielding fragment cations [40], we compare the present results to the thresholds determined in their work. The threshold values listed in Table 2 indicate that our results obtained by electron ionization experiments show in most cases slightly higher appearance energy values than those measured by Cartoni et al. [40] and Bolognesi et al. [39]. We also note that some of the dissociation channels of nitromidazole radical cations were also investigated in our previous work [34].

**Table 2:** Summary of Observed Cations Upon Electron Ionization of 2-Nitroimidazole, Including *m*/*z* Value, Assigned Cation, and Experimental and Calculated Ionization and Appearance Energy Values

*m*/*z*	Assignment		IE and AE values (eV)			
	Cation	Neutral				
			Present exp.	Present calc.	Previous VUV^a^	Previous calc.^a^
28	HCNH^+^	NO + HCN + CO	11.44 ± 0.05	–	11.16 ± 0.06	11.37
30	NO^+^	C_3_N_2_H_3_O	11.11 ± 0.04	–	10.94 ± 0.03	10.60
40	HCCNH^+^	NO_2_ + HCN	13.74 ± 0.03	–	13.8 ± 0.1	13.69*
56	HNCHCO^+^	NO + HCN	11.34 ± 0.09	–	11.14 ± 0.06	11.37
67	C_3_H_3_N_2_^+^	NO_2_	13.00 ± 0.05	12.77*; 12.92	12.76 ± 0.06	11.64*, 12.09
		NO + O	16.29±0.07	16.22		–
83	C_3_H_3_N_2_O^+^	NO	11.12 ± 0.02	–	10.86 ± 0.2	10.60
97	C_3_H_3_N_3_O^+^	O	13.92 ± 0.05	–	13.9 ± 0.2	13.89
113	2NI^+^		9.70 ± 0.02	9.74 (AIE)	9.54 ± 0.01	9.70

*Calculated value refers to the triplet state of respective cation

^a^Refers to experimental and theoretical values reported in reference [40]

Like for imidazole, the parent cation forms the most intense channel upon electron ionization. The ionization energy of 2NI is determined to be 9.70 ± 0.02 eV (see Figure 7i). The calculated values are 9.74 eV for the AIE and 9.99 eV for the vertical ionization energy. Similarly to the case of IMI, the measured value is close to the calculated AIE. However, it should be noted that our theoretical method may overestimate ionization potentials. Both the B3LYP calculated IE and the measured value by Cartoni et al. were reported at 9.35 eV and 9.54 ± 0.01 eV, respectively [40]. These authors determined the IE using the outer valence Green’s functions (OVGF) and obtained an IE value of 9.70 eV which matches perfectly well our experimental result. Since the OVGF method represents the vertical ionization energy (IE_v_), the present results show that during electron ionization no relaxation of the molecules occurs, thus requiring the IE_v_ for ionization. Regarding the fragmentation channels, one pair of complementary fragments is present: The *m*/*z* 83 cation is assigned to C_3_H_3_N_2_O^+^ and thus formed by the loss of a neutral NO group while *m*/*z* 30 is the charged NO group with a neutral C_3_H_3_N_2_O. Bolognesi et al. calculated the fragmentation pathway and showed that both fragment cations are produced via the same transition state with a threshold of 10.60 eV [39]. For the present EI experiment, we determined a fragmentation onset of 11.12 ± 0.02 eV and 11.11 ± 0.04 eV for *m*/*z* 83 and 30, respectively (see Figures 7g, c), while Cartoni et al. reported 10.86 ± 0.02 eV and 10.94 ± 0.03 eV [40]. Taking our measured IE value of 9.70 ± 0.02 eV and the TS barrier of 1.37 eV [39], which agrees with the barrier calculated in a previous work [34], we obtain the AE of 11.07 eV, in excellent agreement with our experimental finding. The fragment ion at *m*/*z* 83 C_3_H_3_N_2_O^+^ further decomposes into two other fragments observed in the mass spectrum, the *m*/*z* 56 and the *m*/*z* 28 cations. The detailed description of the fragmentation pathway can be found in [40]. Basically, the imidazole ring breaks at the N1–C2 position and multiple hydrogen transfers occur until finally the hydrogen cyanide molecule, HCN, is released from the complex, forming HNC(H)CO^+^ at *m*/*z* 56. The calculated threshold for the reaction is defined by the energetically highest transition state of 11.37 eV. This cation can even fragment further under the release of carbon monoxide, CO, to yield HNCH^+^ at *m*/*z* 28. As no higher transition states are involved, the same threshold value holds. Our experimental results are 11.34 ± 0.09 eV and 11.44 ± 0.05 eV, for *m*/*z* 56 and 28, respectively (see Figures 7e, b) that support the predictions by Cartoni et al., even though their experimental values of 11.14 ± 0.06 eV and 11.16 ± 0.06 eV are slightly lower [40].

At *m*/*z* 97, we observe C_3_H_3_N_3_O^+^, the cation formed under the release of an atomic oxygen. We determine an experimental onset of 13.92 ± 0.05 eV (see Figure 7h), which matches the experimental value determined by Cartoni et al. of 13.9 ± 0.2 eV [40]. In their calculations, Cartoni et al. considered different electronic configurations for the pair of cation and neutral fragment formed. It turned out that only for the doublet state of C_3_H_3_N_3_O^+^ and the singlet state of atomic oxygen, the obtained AE of 13.89 eV agrees energetically with the experiments.

**Figure 8:**
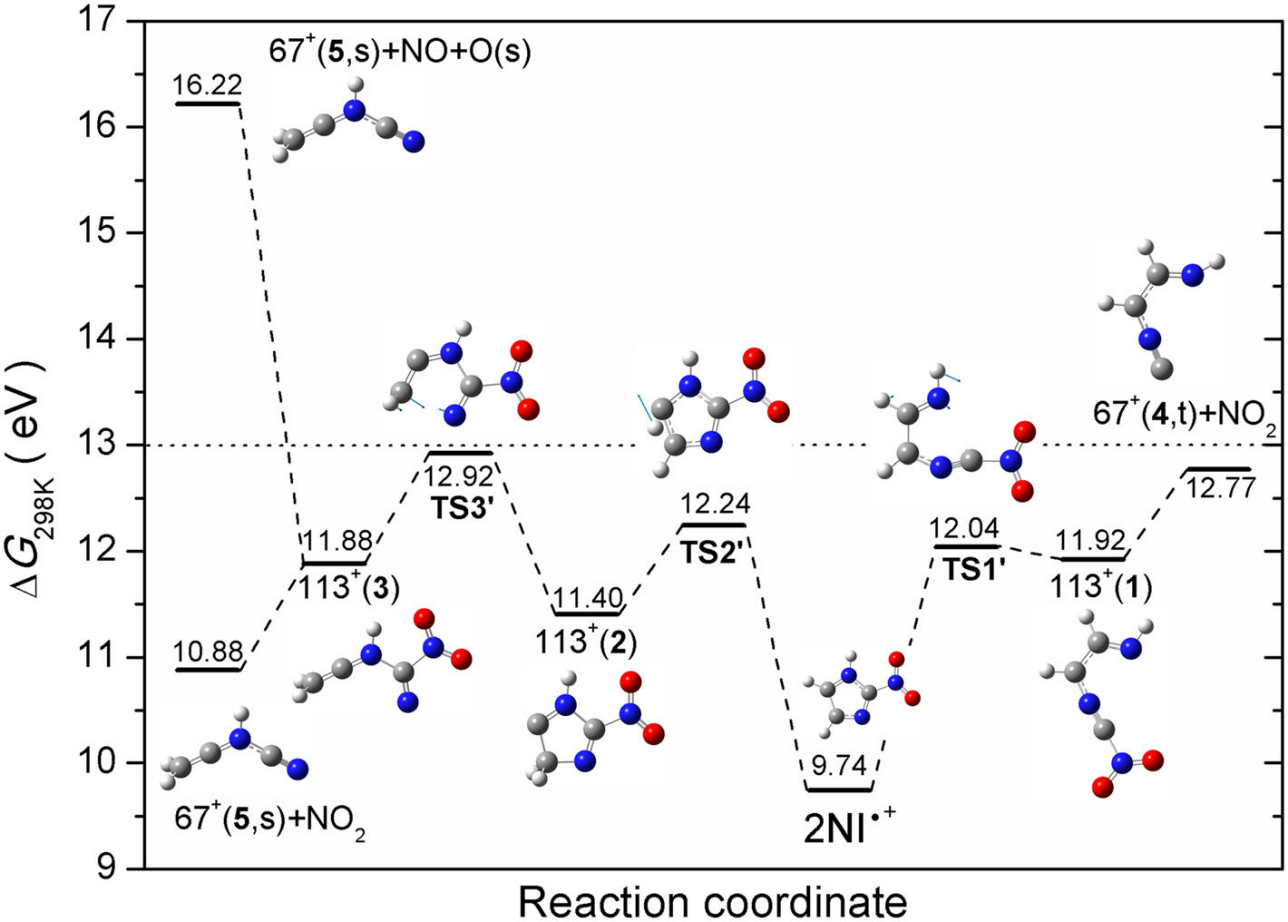
M06-2x/aug-cc-PVTZ calculated potential energy diagram for the decomposition of the 2NI^+^ leading to the formation of fragment ion at *m*/*z* 67. The blue arrows in the respective TS show the displacement vectors. The resulting cation formed through loss of NO_2_ group, 67^+^(**4**), is in the triplet state, while 67^+^(**5**) is in the singlet state

Two fragmentation pathways with relatively high appearance energies lead to the formation of cations *m*/*z* 67 and *m*/*z* 40. Additionally, *m*/*z* 40 features the most intense ion yield among all the fragment ions (see Figure 2). Since both cations are also observed in the EI experiments with imidazole, as discussed above, and in previous studies [26, 28], it is straight forward to explain the formation of *m*/*z* 40 from the dehydrogenated imidazole at *m*/*z* 67, C_3_H_3_N_2_^+^. However, the pathway yielding *m*/*z* 67 for 2NI must be different from IMI, as formation from 2NI proceeds through the loss of the –NO_2_ group from the C2 position, which is not the preferential position for the H loss from IMI^+^. The likely loss of HCN from *m*/*z* 67 was computationally investigated by Cartoni et al. who further differentiated between the singlet and triplet states of C_3_H_3_N_2_^+^. Based on the determined experimental AE values of 13.8 ± 0.1 eV for the fragment at *m*/*z* 40, they were able to exclude the singlet state of C_3_H_3_N_2_^+^ with a calculated onset of 15.16 eV and verified the fragmentation pathway via the triplet state with a threshold of 13.69 eV [40]. The AE determined with our EI experiment is 13.74 ± 0.03 eV (see Figure 7d) and, thus, supports Cartoni et al.’s experimental AE value. The fragmentation pathway for *m*/*z* 40 was calculated by Cartoni et al. and is summarized here for the triplet state for the sake of completeness. First, the parent cation is ionized and isomerizes before it decays into the NO_2_ group and the charged dehydrogenated imidazole. This is the step where either the singlet or triplet state is formed. In case of the triplet, the ring opens at the N1–C5 position. The according transition state possesses the highest energy, thus defining the AE of the pathway. Afterwards, the H-N1-C2 moiety shifts position to C5, creating a new bond between C2 and C5. Subsequently, the C4–C5 bond breaks giving rise to the *m*/*z* 40 fragment HCCNH^+^ and the neutral HCN. Regarding the *m*/*z* 67 cation the interpretation remained inconclusive in Cartoni et al.’s discussion. Both calculated AE values, 11.64 eV and 12.09 eV for the triplet and singlet state, respectively, were too low to explain their observed onset at 12.76 ± 0.06 eV. We report two experimentally determined threshold values at 13.00 ± 0.05 eV and at 16.29 ± 0.07 eV (see Figure 7f). For the formation of *m*/*z* 67 cation, we suggest that either a hydrogen transfer or a ring opening is required before the release of the NO_2_. As discussed above in the case of the imidazole molecule, simple bond breaking was insufficient to explain the formation of *m*/*z* 67. We propose two pathways for the formation of *m*/*z* 67 shown in Figure 8. The first pathway proceeds through N1–C2 ring opening via the TS1′ of 12.04 eV forming an open chain cation 113^+^(**1**), followed by a release of the NO_2_ and *m*/*z* 67 in the triplet state with a ∆*G* of 12.77 eV that is slightly lower than our AE value, though in excellent agreement with Cartoni et al. [40]. The second pathway proceeds with H transfer from C5 to C4 with TS2′ of 12.24 eV forming the cation 113^+^(**2**); further, opening of the imidazole ring between C4 and N3 through TS3′ of 12.92 eV leads to the formation of the cation 113^+^(**3**). Simple C–NO_2_ bond cleavage results in 67^+^(**5**) in singlet state with ∆*G* of 10.88 eV; however, the apparent AE is given by the TS3′ of 12.92 eV, which is in excellent agreement with present and previous experimental observation. If the cation 113^+^(**3**) dissociates into the same fragment cation 67^+^(**5**), however, with the release of NO and O in the singlet state, the respective free energy of reaction ∆*G* of 16.22 eV is in great agreement with the second AE threshold value (16.29 ± 0.07) measured presently.

## Conclusions

The present study revealed the reactions in imidazole and 2-nitroimidazole upon electron ionization. Since imidazole is a basic building block of life and dedicated derivatives based on the nitroimidazole group are already used as radiosensitizers in radiation therapy and as antibiotics, this knowledge is crucial to understand the basic reactions occurring under electron interactions mainly those yielding ionization processes. The mass spectra of IMI and 2NI show both the parent cation as the peak with highest intensity, revealing that this ion can be efficiently stabilized upon electron interaction at 70 eV. A comparison to earlier studies demonstrated that electron ionization results in a different relative intensity of product ions when compared to the photoionization spectrum by Schwell et al. [28]. Though it may be also related to different experimental conditions, especially the strong parent cation seems to be a characteristic for electron ionization, which agrees with the EI mass spectrum of Klebe et al. [26]. The observed dissociation channels in IMI yield cations at *m*/*z* 67, 41, 40, and 28, which we were able to assign to different structures in agreement with the experimentally measured thresholds. It was shown that *m*/*z* 67, [IMI − H]^+^, cannot be formed in a direct loss of H through single bond breaking. Instead, upon ionization of the IMI molecule, an H atom can transfer between various positions of the ring, and further, after ring opening H is lost from different positions with preference for C4 and C5, as suggested from earlier work. Most of the H transfer reactions lie between 11.22 and 11.80 eV, at which electron energy three fragments of IMI appear, namely *m*/*z* 67, *m*/*z* 41, and *m*/*z* 28. Thus, some of the transition states are involved in the formation of several ions. We assigned three different ions to the AEs derived for *m*/*z* 67, three for *m*/*z* 41, and only in the case of the fragment at *m*/*z* 28, it is assigned to a single structure HCNH^+^. Compared with the assignments from previous PI studies, we predict that more fragmentation pathways are opened. We observed the general trend that in the present study, the AE values are slightly higher than in previous PI studies. The only fragment cation with a mentionable deviation is *m*/*z* 40. So far, only Schwell et al. [28] reported this cation before at a threshold 1 eV lower than determined by the present EI study. We ascribe this deviation to different dissociation pathways.

We also note that systematically lower values were obtained in comparison to the previous EI values by Klebe et al. [26]. This deviation may be explained by the increased precision of the electron energy scale due to the utilization of an electron monochromator [53] in our study. 2NI exhibits similar fragment cations to IMI, such as, *m*/*z* 67 C_3_H_3_N_2_^+^, 40 HNCCH^+^, and 28 HCNH^+^. However, they are formed through different dissociation pathways due to the presence of the NO_2_ group at the C2 position of the imidazole ring. Additionally, fragment cations are observed with *m*/*z* 97, 83, 56, and 30. These are assigned to the cations C_3_H_3_N_3_O^+^ due to O loss from 2NI and C_3_H_3_N_2_O^+^ due to NO loss from 2NI, HNCHCO^+^, and NO^+^, respectively. For 2NI, the fragment cations with *m*/*z* 40, 30, and 28 have the highest ion yields. As for IMI, our determined AE values are usually slightly higher than those from PI study. The 2NI dissociation mechanisms were proposed in the earlier photoionization study for all observed cations, with the exception of the formation of ion at *m*/*z* 67 formed through the loss of the NO_2_ group, where proposed pathways did not match the experimentally observed AE threshold value and remained inconclusive. We propose two novel pathways leading to the AE observed in both electron and photon ionization. First pathway involves the ring opening reaction followed by the release of NO_2_ and the formation of the *m*/*z* 67 cation in the triplet state. Second pathway involves H transfer reaction followed by a ring opening and the release of NO_2_ forming the *m*/*z* 67 cation in the singlet state. Additionally, electron ionization showed two thresholds, while PI only one. The second threshold observed in the present study can be associated with the latter pathway proposed forming the *m*/*z* 67 cation in the singlet state with neutrals NO and O in the singlet state. The main characteristics with radiosensitizing impact of 2NI at the molecular level observed here are the stabilized parent cation and the release of NO as reactive nitrogen species.

## Electronic Supplementary Material

ESM 1 (PDF 1555 kb)
