# Supplementary material for: Electron Ionization of Imidazole and Its Derivative 2-Nitroimidazole
Source: J Am Soc Mass Spectrom. 2019 Oct 30;30(12):2678–91. doi: 10.1007/s13361-019-02337-w (PMC6914720; doi:10.1007/s13361-019-02337-w)
Supplement: Supplementary file 1 — (PDF 1555 kb) [file 13361_2019_2337_MOESM1_ESM.pdf]

Electronic Supplementary Material

Electron Ionization of Imidazole and its Derivative 2-Nitroimidazole

Rebecca Meißner,[1,2] Anita Ribar,[1] Katharina Fink,[1] Linda Feketeová,[1,3] Stephan Denifl[1]

[1] Institut für Ionenphysik und Angewandte Physik Physik and Center for Molecular Biosciences Innsbruck (CMBI), Universität Innsbruck, Technikerstraße 25, 6020 Innsbruck, Austria
[2] Atomic and Molecular Collisions Laboratory, CEFITEC, Department of Physics, Universidade NOVA de Lisboa, 2829-516 Caparica, Portugal
[3] Institut de Physique Nucléaire de Lyon; CNRS/IN2P3, UMR5822, Université de Lyon, Université Claude Bernard Lyon 1, 43 Bd du 11 novembre 1918, 69622 Villeurbanne, France

rebecca.meissner@uibk.ac.at; stephan.denifl@uibk.ac.at; l.feketeova@ipnl.in2p3.fr

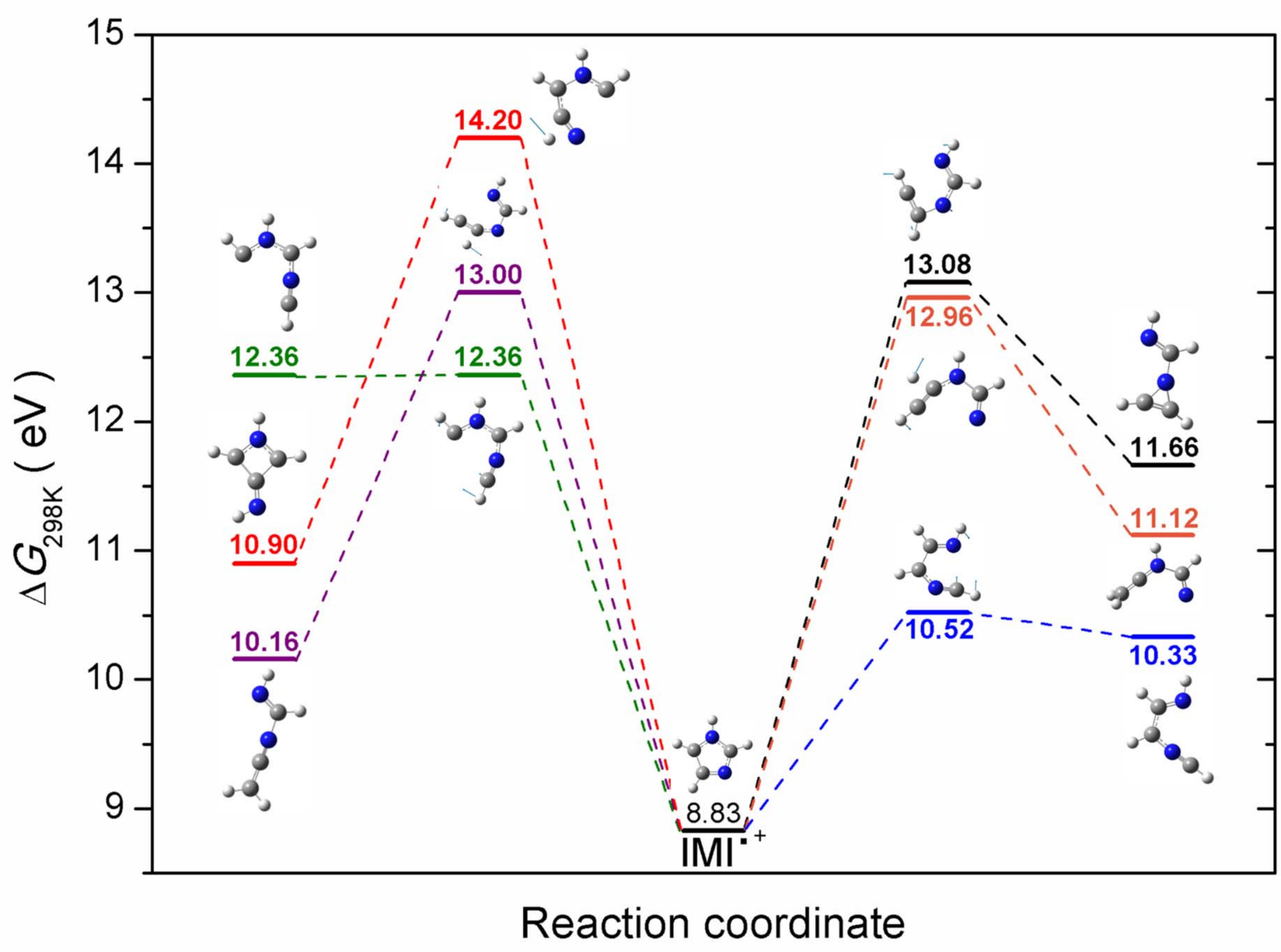

Figure ESM_1: M06-2x/aug-cc-PVTZ calculated potential energy diagram for ring opening reactions in the ionized IMI^{+}. The Free reaction energy Δ*G* shown in eV. The blue arrows in the respective TS show the displacement vectors. Some of the ring opening reactions are associated with concomitant H transfer.

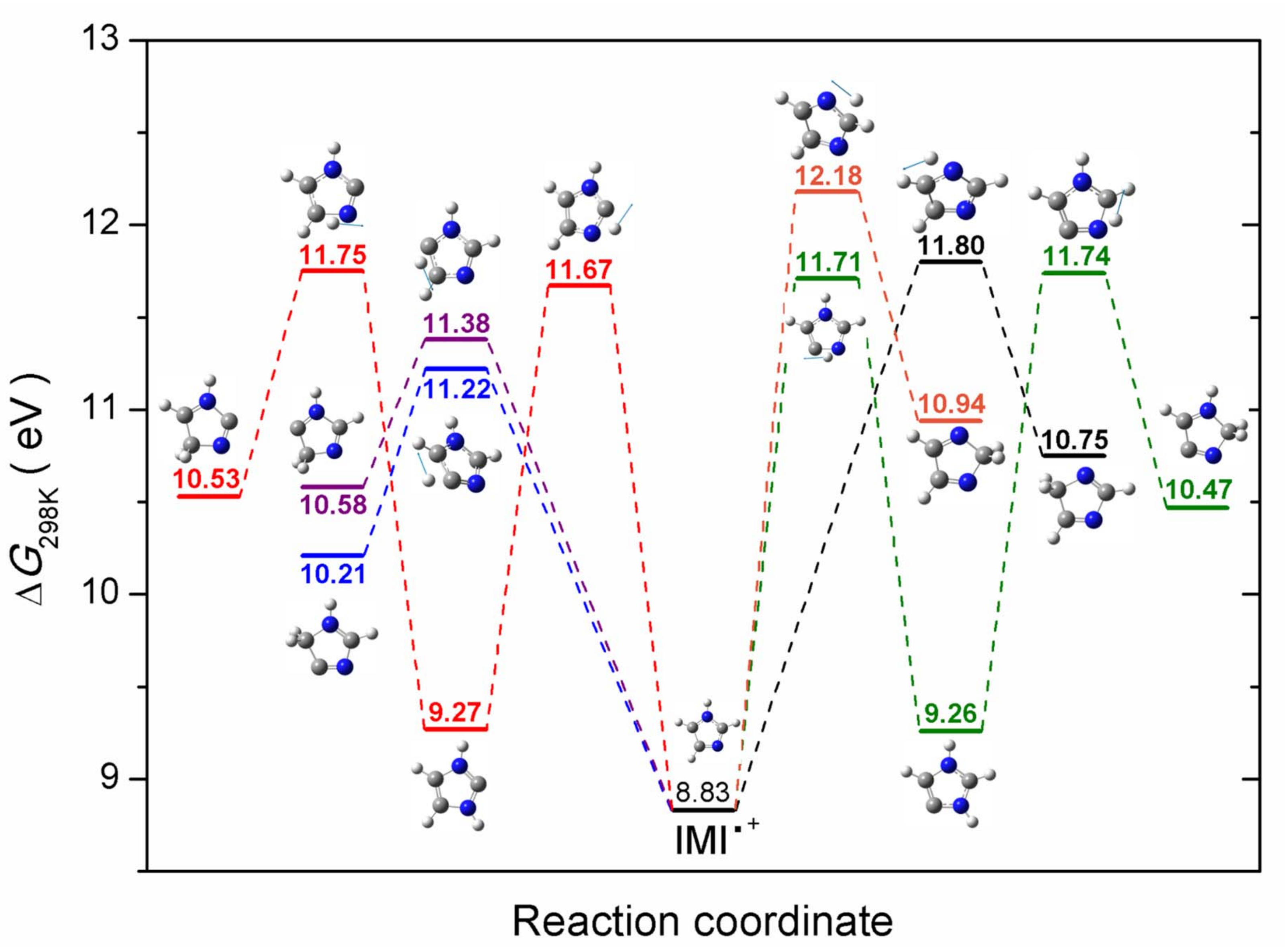

Figure ESM_2: M06-2x/aug-cc-PVTZ calculated potential energy diagram for H transfer reactions in the ionized IMI^{+}. The Free reaction energy Δ*G* shown in eV. The blue arrows in the respective TS show the displacement vectors.

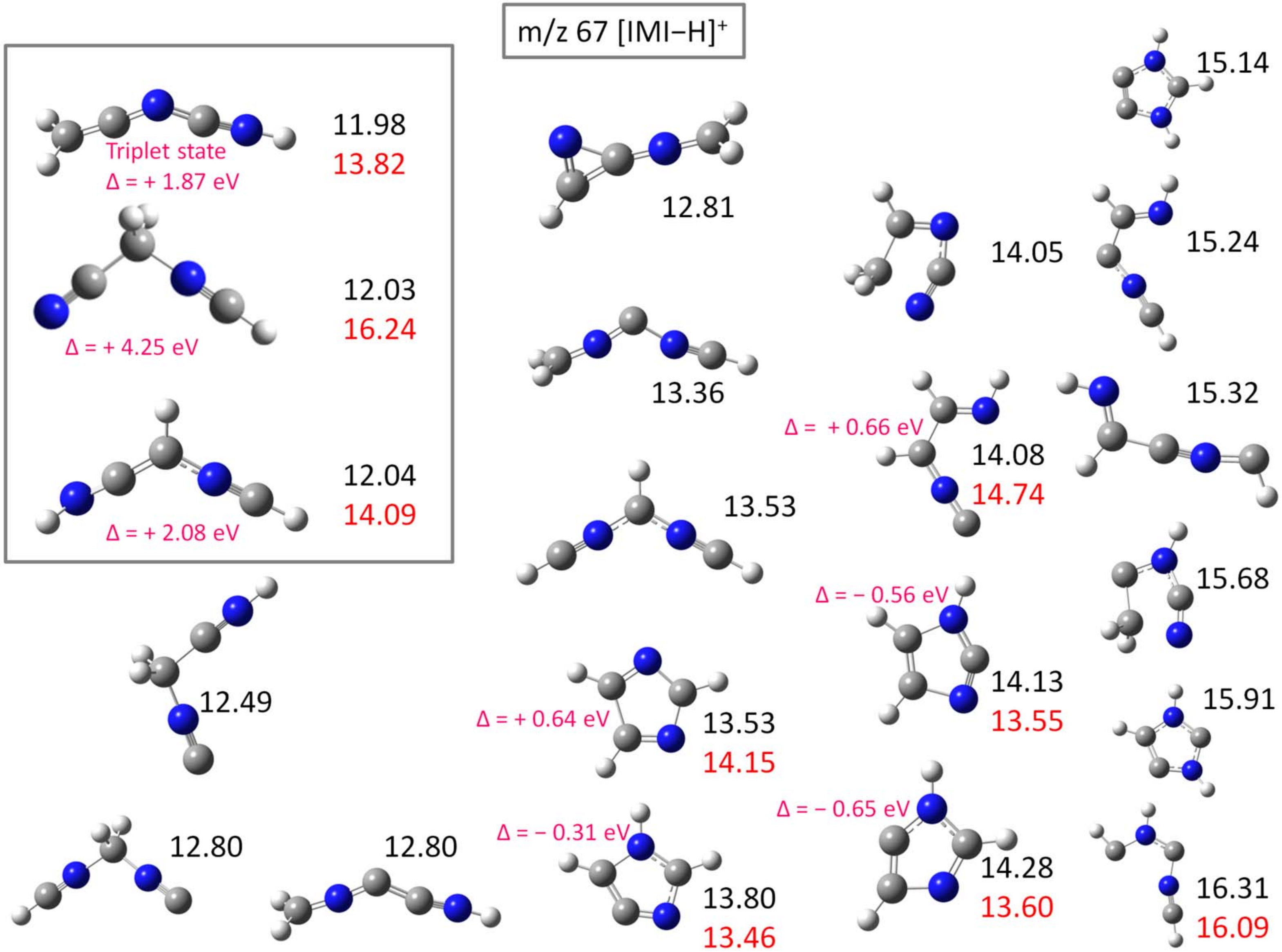

Figure ESM_3: M06-2x/aug-cc-PVTZ calculated structures of fragment ion at m/z 67 $[IMI{-}H]^+$. The numbers in black are associated Free reaction energies ΔG shown in eV, while the numbers in red refer to triplet states of $[IMI{-}H]^+$.

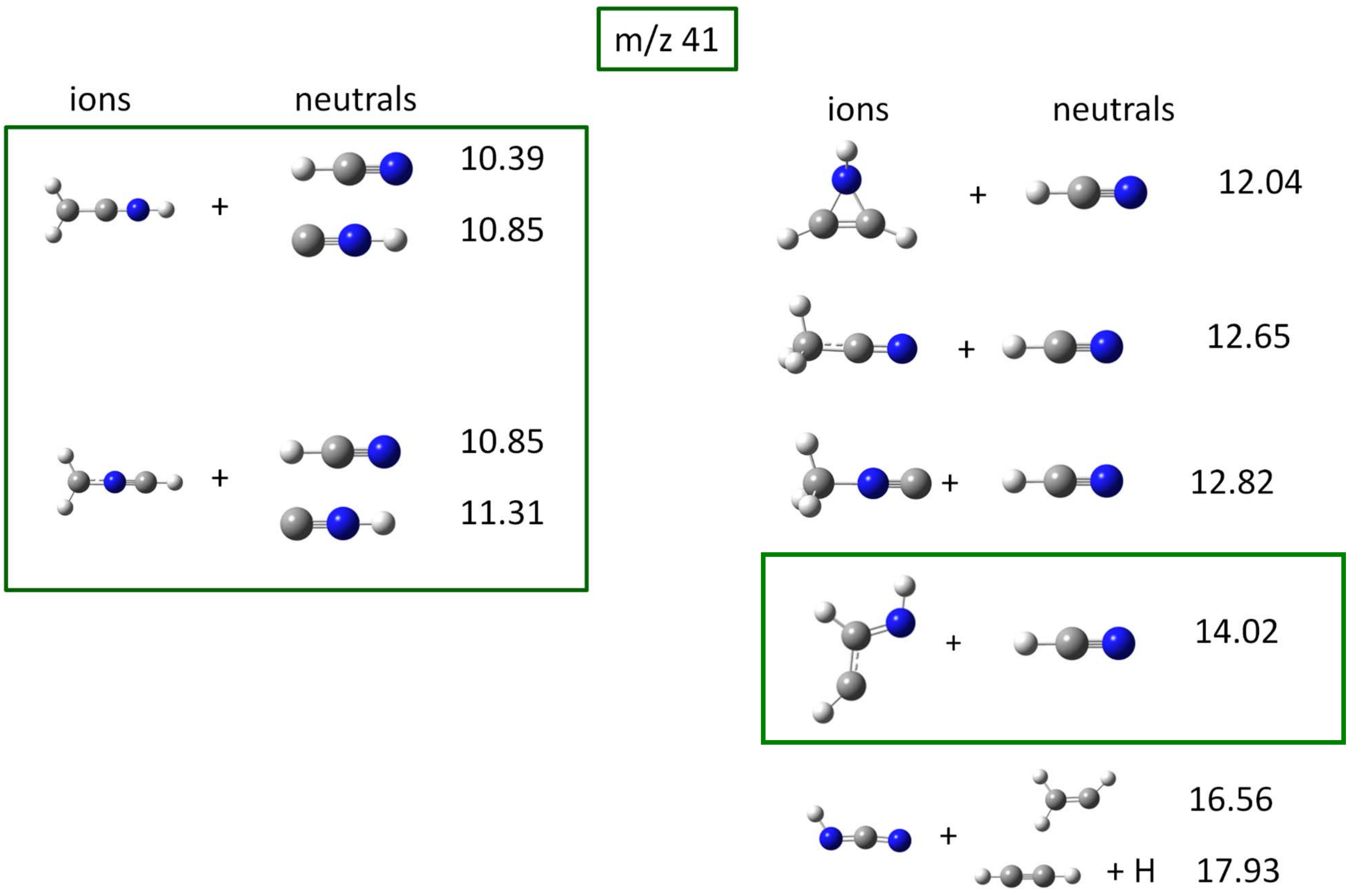

Figure ESM_4: M06-2x/aug-cc-PVTZ calculated structures of fragment ion at m/z 41. The numbers are associated Free reaction energies Δ*G* shown in eV.

m/z 40

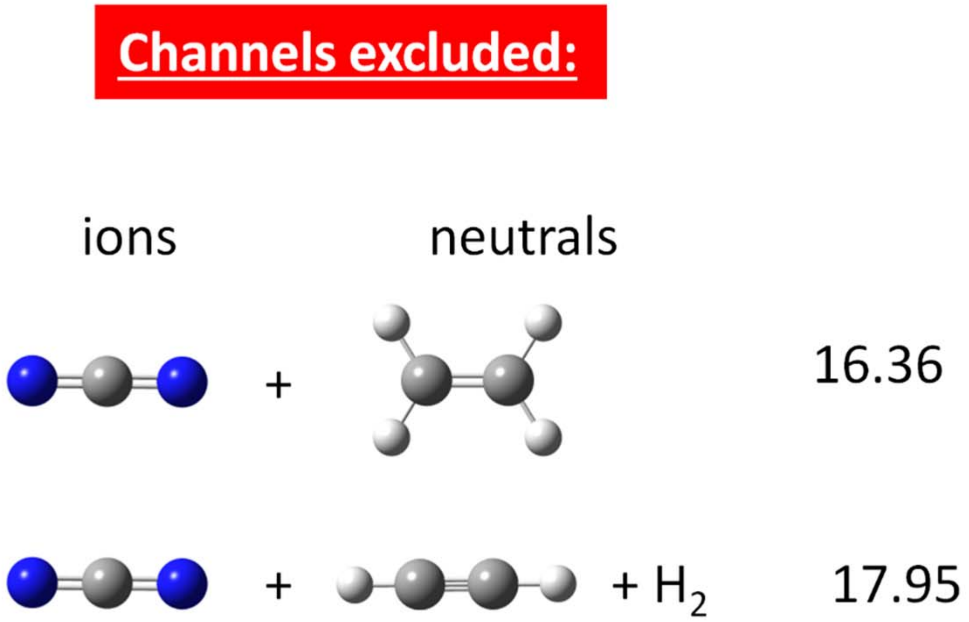

Figure ESM_5: M06-2x/aug-cc-PVTZ calculated structures of fragment ion at m/z 41. The numbers are associated Free reaction energies Δ*G* shown in eV. These are excluded channels due to the high Δ*G*.

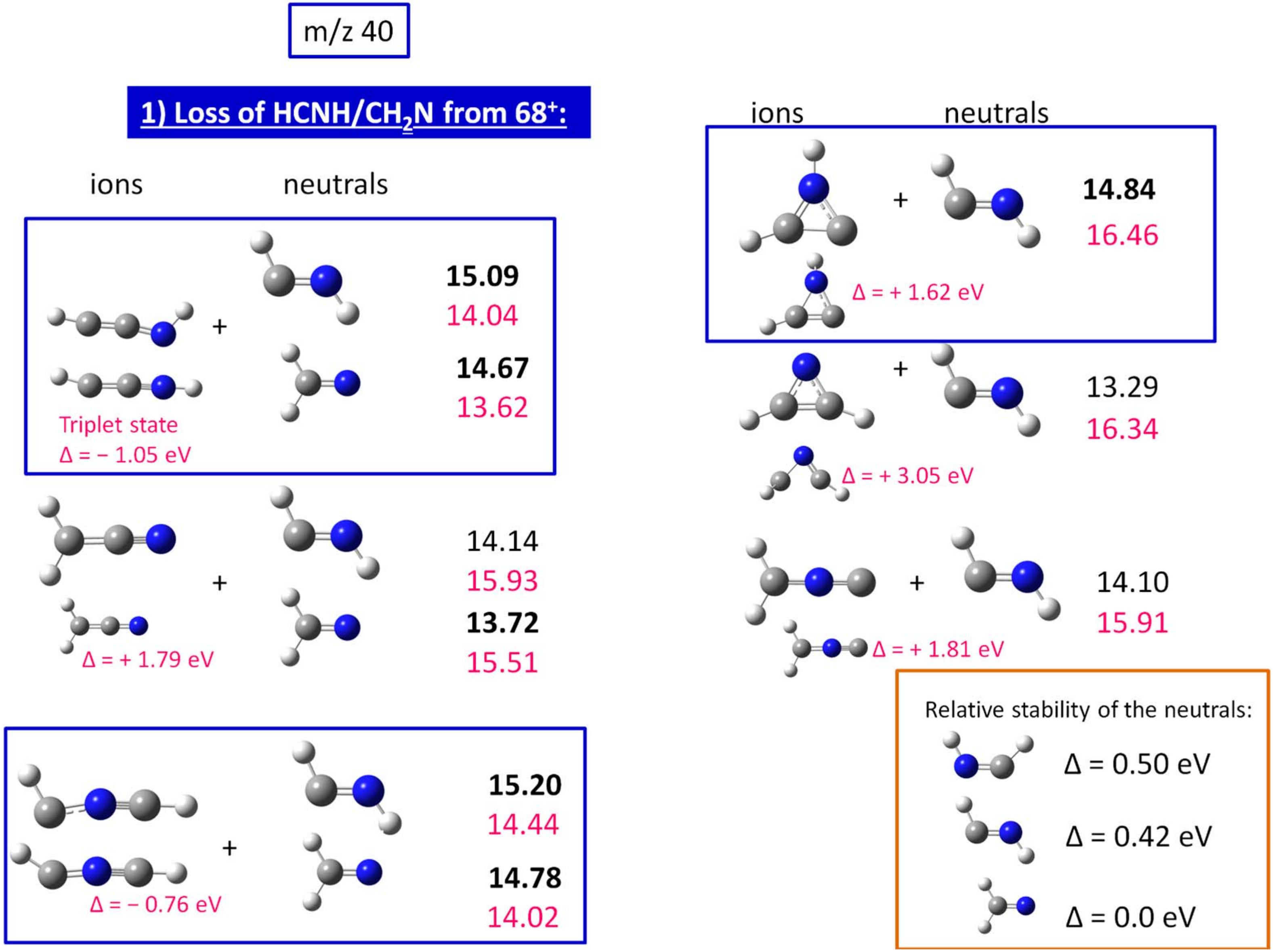

Figure ESM_6: M06-2x/aug-cc-PVTZ calculated structures of fragment ion at m/z 40 considering the dissociation pathway via loss of a single neutral and the relative stabilities of the neutrals. The numbers in black are associated Free reaction energies Δ*G* shown in eV, while the red ones refer to the triplet states.

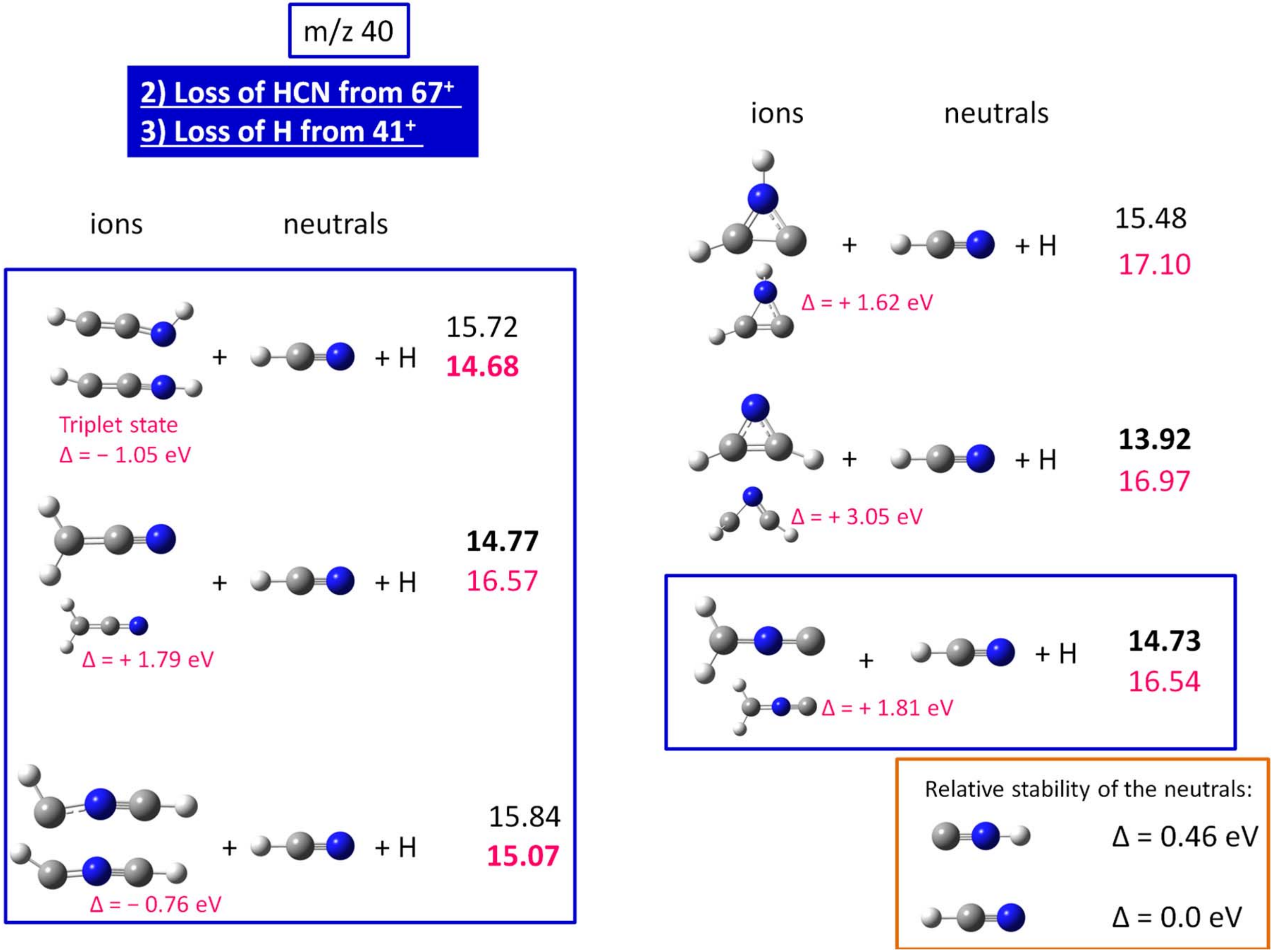

Figure ESM_7: M06-2x/aug-cc-PVTZ calculated structures of fragment ion at m/z 40 considering the dissociation pathways via either loss of HCN from IMI^{+}, or via loss of H from 41^{+}. The numbers in black are associated Free reaction energies Δ*G* shown in eV, while the red ones refer to the triplet states.

1) Loss of HCNH/CH_2N from 68⁺:

m/z 40

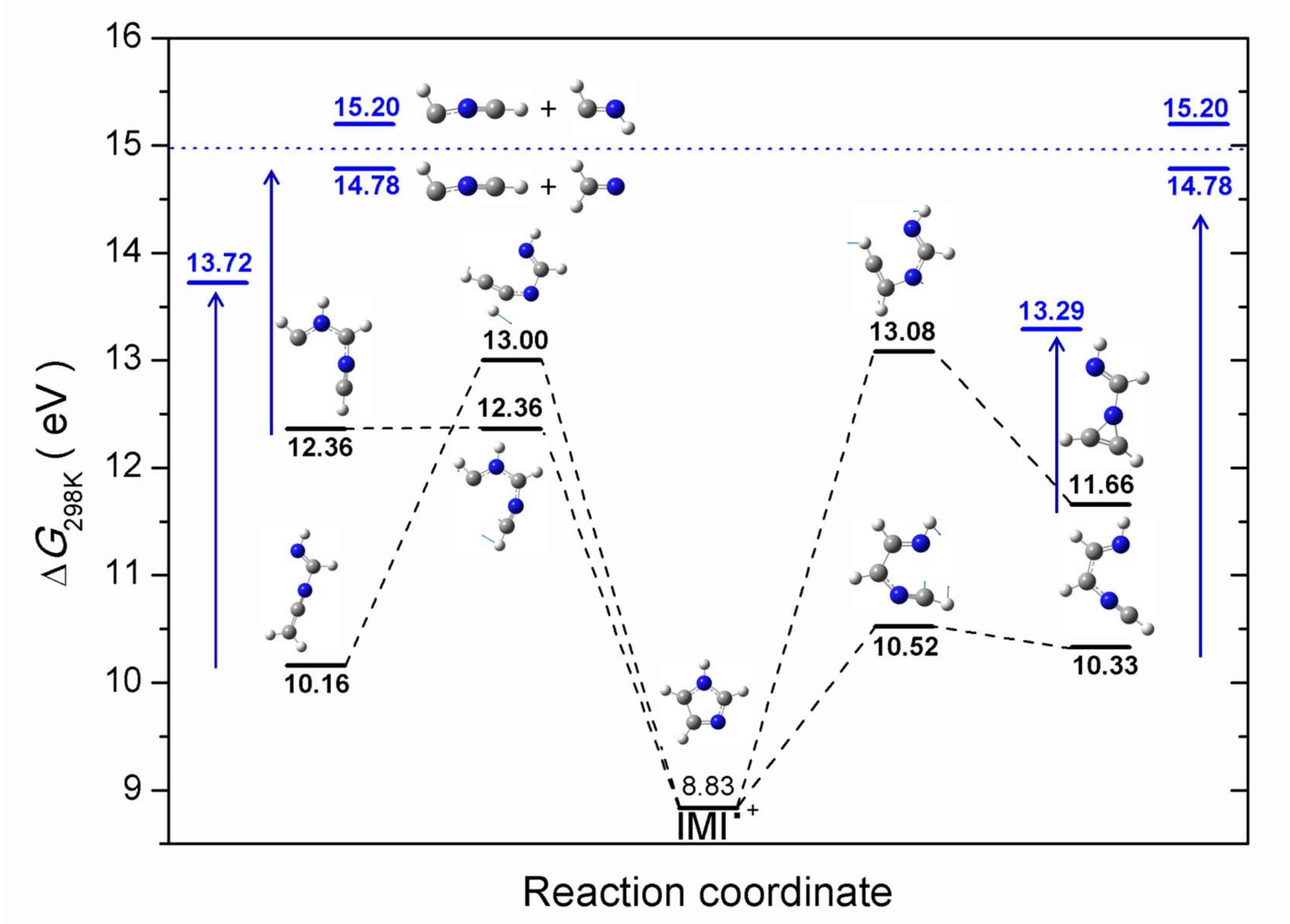

Figure ESM_8: M06-2x/aug-cc-PVTZ calculated potential energy diagram for the formation of the fragment ion at m/z 40 considering the dissociation pathway via loss of a single neutral. The Free reaction energy Δ*G* shown in eV. The blue arrows in the respective TS show the displacement vectors. The dotted line corresponds to the experimental value.

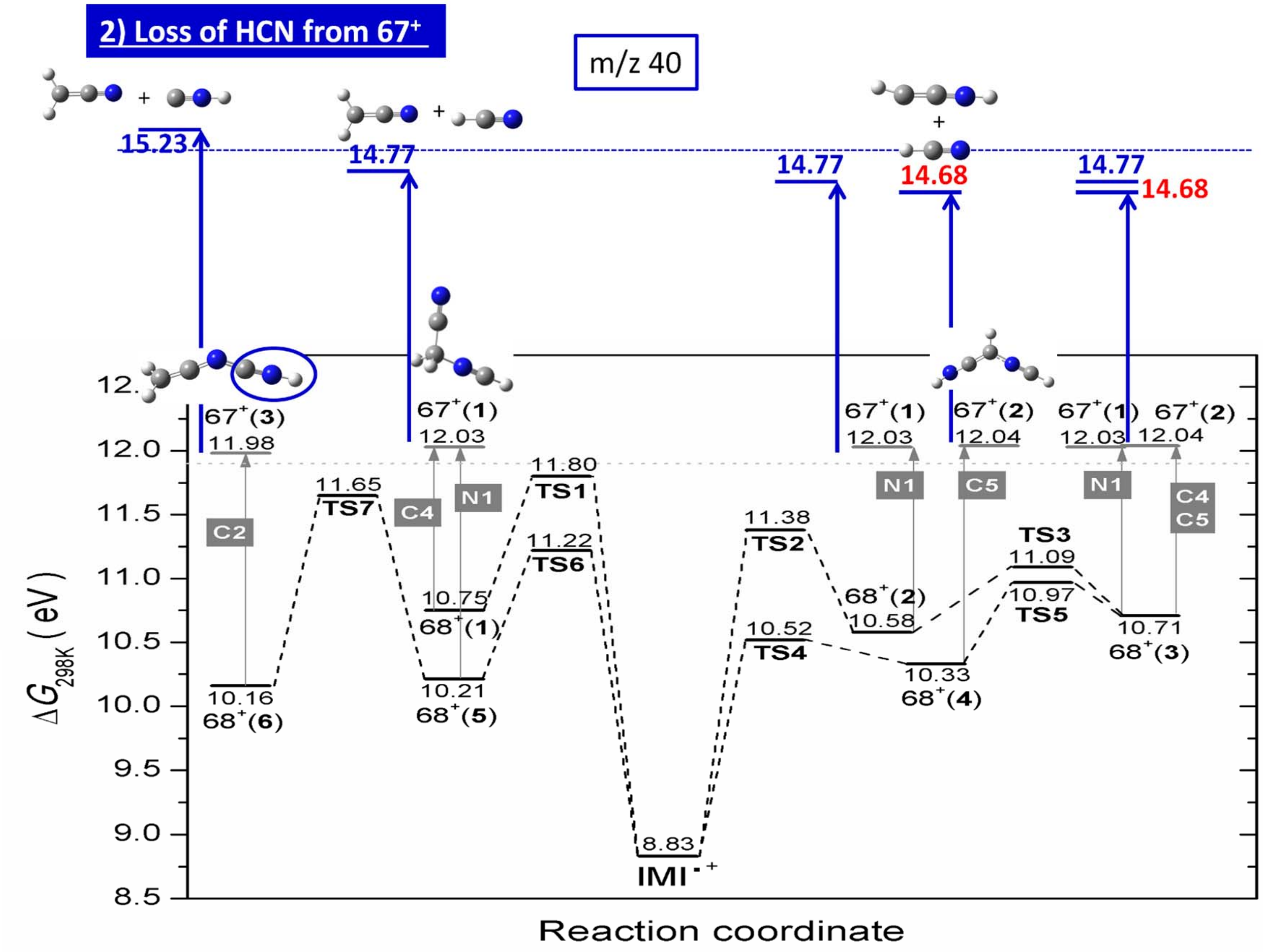

Figure ESM_9: M06-2x/aug-cc-PVTZ calculated potential energy diagram for the formation of the fragment ion at m/z 40 considering the dissociation pathways via the loss of HCN from [IMI−H]$^+$. The energy scale in blue is schematic. The Free reaction energy Δ*G* shown in eV. The dotted line represents the experimental value.

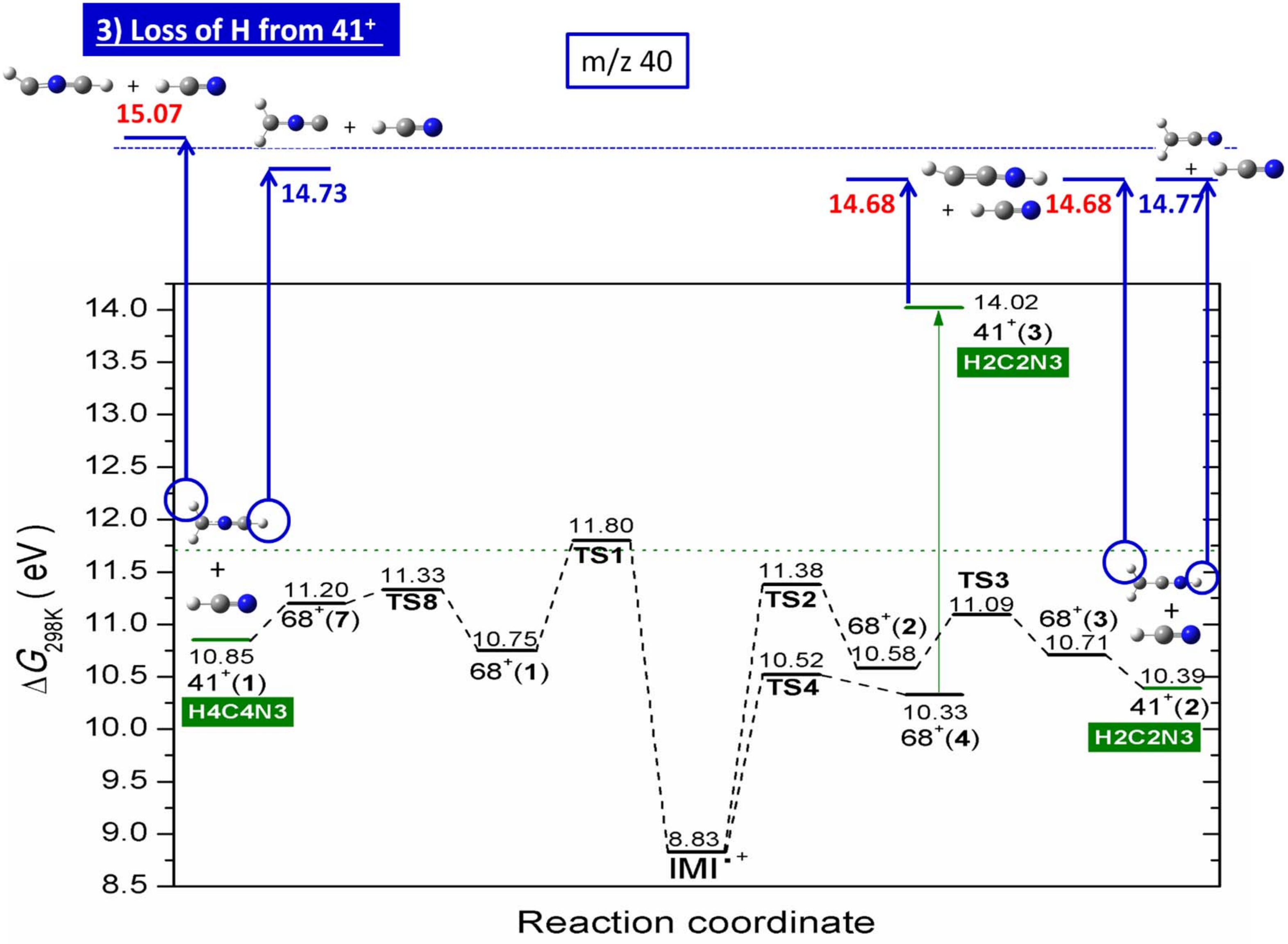

Figure ESM_10: M06-2x/aug-cc-PVTZ calculated potential energy diagram for the formation of the fragment ion at m/z 40 considering the dissociation pathways via the loss of H from 41^+. The energy scale in blue is schematic. The Free reaction energy Δ*G* shown in eV. The dotted line represents the experimental value.

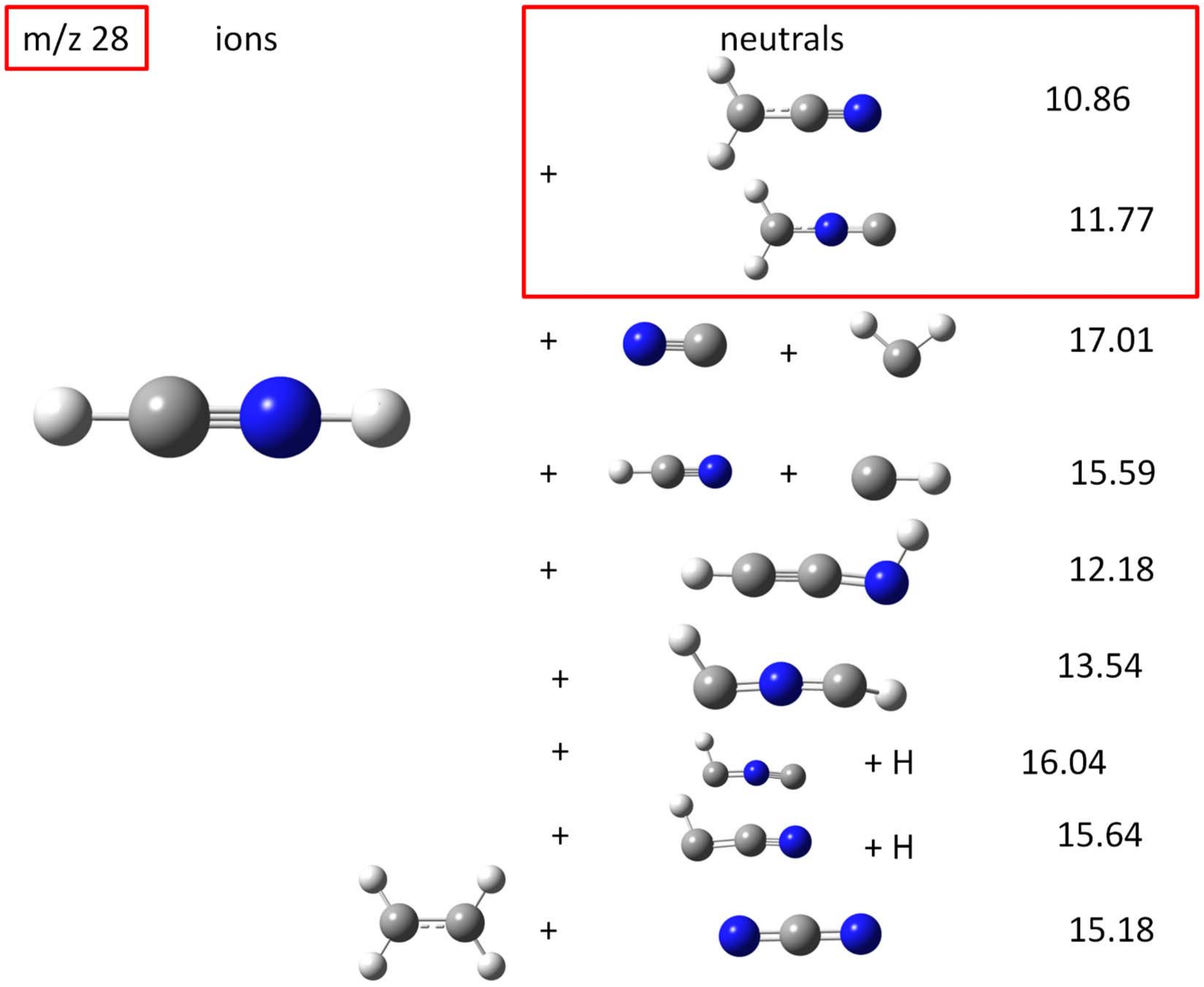

Figure ESM_11: M06-2x/aug-cc-PVTZ calculated structures of fragment ion at m/z 28. The numbers are associated Free reaction energies Δ*G* shown in eV.

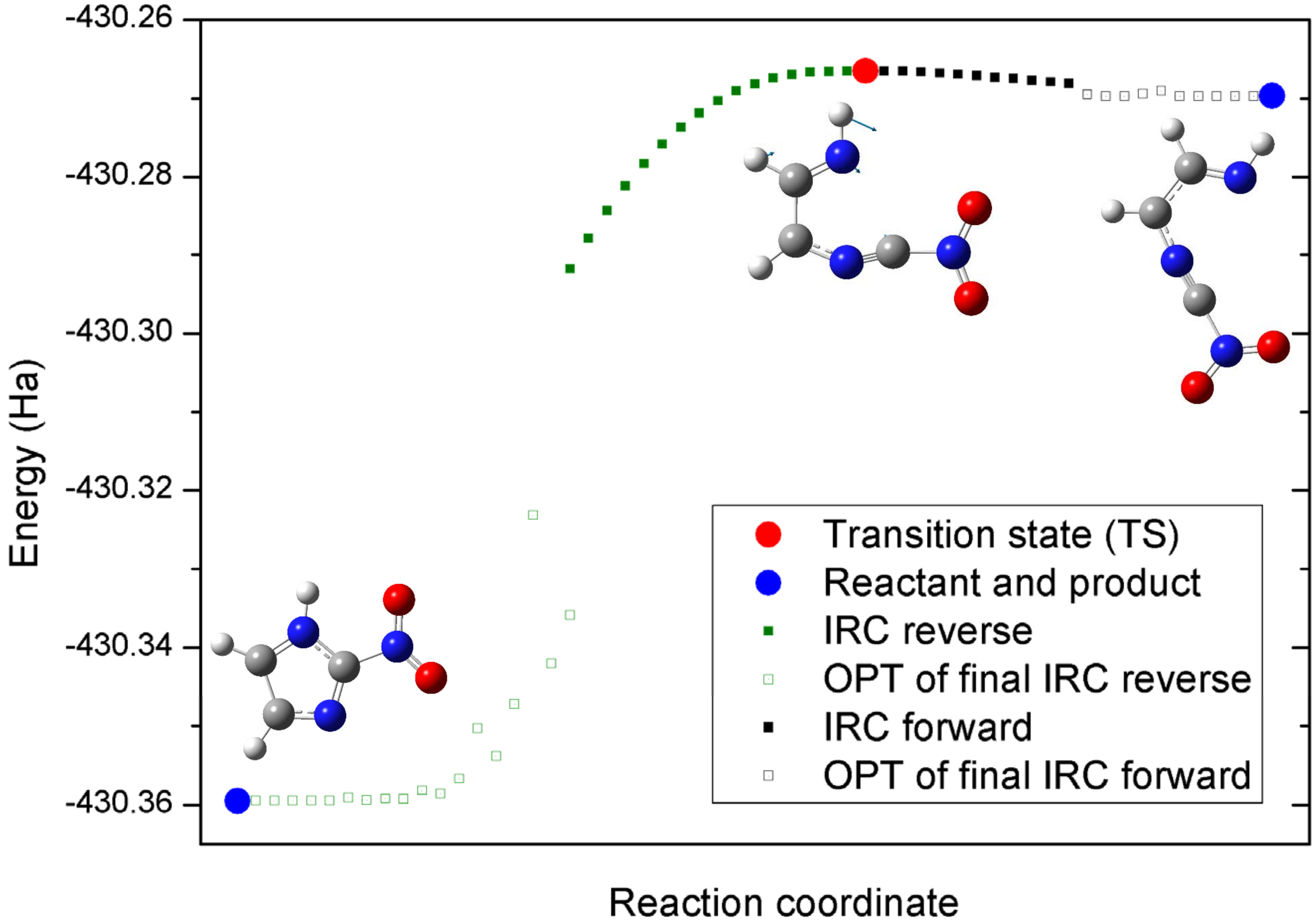

Figure ESM_12: M06-2x/aug-cc-PVTZ calculated energies (in Hartrees) showing: TS (red circle) for the ring opening reaction involved in the dissociation pathway associated with NO_2 loss; Reactant (2NI) and product (blue circles); IRC reverse calculation (green full squares); Optimization of the final IRC reverse structure (green empty squares); IRC forward calculation (black full squares); Optimization of the final IRC forward structure (black empty squares). Associated structures with reactant (2NI), TS, and product (open chain 2NI) are also shown.
